# Rational design of heterogenized molecular phthalocyanine hybrid single-atom electrocatalyst towards two-electron oxygen reduction

**DOI:** 10.1038/s41467-023-37066-y

**Published:** 2023-03-14

**Authors:** Wenjun Fan, Zhiyao Duan, Wei Liu, Rashid Mehmood, Jiating Qu, Yucheng Cao, Xiangyang Guo, Jun Zhong, Fuxiang Zhang

**Affiliations:** 1 State Key Laboratory of Catalysis, Dalian National Laboratory for Clean Energy, Dalian Institute of Chemical Physics, Chinese Academy of Sciences, 116023 Dalian, China; 2 State Key Laboratory of Solidification Processing, School of Materials Science and Engineering, Northwestern Polytechnical University, 710072 Xi’an, P. R. China; 3 State Key Laboratory of Fine Chemicals, School of Chemical Engineering, Dalian University of Technology, 116024 Dalian, China; 4 University of Chinese Academy of Sciences, 100049 Beijing, China; 5 Institute of Functional Nano and Soft Materials (FUNSOM), Jiangsu Key Laboratory for Carbon-Based Functional Materials and Devices, Soochow University, 215123 Suzhou, Jiangsu P. R. China

**Keywords:** Electrocatalysis, Electrocatalysis, Electrocatalysis, Electrocatalysis

## Abstract

Single-atom catalysts supported on solid substrates have inspired extensive interest, but the rational design of high-efficiency single-atom catalysts is still plagued by ambiguous structure determination of active sites and its local support effect. Here, we report hybrid single-atom catalysts by an axial coordination linkage of molecular cobalt phthalocyanine with carbon nanotubes for selective oxygen reduction reaction by screening from a series of metal phthalocyanines via preferential density-functional theory calculations. Different from conventional heterogeneous single-atom catalysts, the hybrid single-atom catalysts are proven to facilitate rational screening of target catalysts as well as understanding of its underlying oxygen reduction reaction mechanism due to its well-defined active site structure and clear coordination linkage in the hybrid single-atom catalysts. Consequently, the optimized Co hybrid single-atom catalysts exhibit improved 2e^−^ oxygen reduction reaction performance compared to the corresponding homogeneous molecular catalyst in terms of activity and selectivity. When prepared as an air cathode in an air-breathing flow cell device, the optimized hybrid catalysts enable the oxygen reduction reaction at 300 mA cm^−2^ exhibiting a stable Faradaic efficiency exceeding 90% for 25 h.

## Abstract (web summary)

Difficulties in elucidating active sites and the role of the support hamper the development of high-efficiency two-electron oxygen reduction electrocatalysts. Here, the authors develop hybrid single-atom catalysts by rational experimental and theoretical screening for hydrogen peroxide production.

## Introduction

The development of single-atom catalysts (SACs) with active metal monodispersed on solid substrates has sparked extensive interest in promoting the catalytic performance of various reactions^1–3^, particularly in the community of energy-related electrocatalysis^4^. In comparison to their bulk or nanosized equivalents, they display maximum atom utilization efficiency and unconventional catalytic activities due to their unique atomic structures and electronic properties^5–8^. It has also been theoretically demonstrated that SACs with a well-defined single atomic site are highly favorable for the precise understanding of the catalytic mechanism and rational design of catalysts^7,9,10^. However, most SACs have been synthesized experimentally at high temperatures by strong covalent interactions or metal-support interactions to embed active metals in a variety of supports^2,11,12^. In this case, it remains actual difficulty in controlling and confirming the exact atomic structure of the active sites in SACs. The oxygen reduction reaction (ORR) electrocatalysis, which serves as the crucial half-reaction of fuel cells (four electrons) or the ideal process for green synthesis of hydrogen peroxide (two electrons), has received intense attention. As a promising candidate, carbon-supported SACs with atomic transition-metal-heteroatoms moieties have been extensively investigated. Nevertheless, they have been mostly prepared by pyrolysis of metal-, heteroatom- and carbon-containing precursors, resulting in structural and compositional heterogeneity^7,13–17^, rendering great difficulties in the definitive correlation between active site structures and catalytic performances as well as the rational design of new generation of SACs.

Different from the heterogeneous catalysts^18^, homogeneous molecular catalysts are composed of definite atomic structures and adjustable coordination environment/single atomic sites, allowing people to systematically tailor the catalytic activity and selectivity^19–21^. Molecular catalysts, on the other hand, are generally subjected to problematic separation, easy aggregation, and poor conductivity^22,23^. To address those flaws, strategies and methodologies for heterogenization of the molecular catalysts have been developed^24,25^. Meanwhile, the linkage between catalyst and support has been shown to have a significant impact on catalytic activity and selectivity. For example, the π-π interactions between carbon support and molecular catalyst can regulate the binding energy of intermediates, resulting in improved selectivity and activity^26–28^; covalent grafting with a sulfoxide dopant was found to increase the turnover frequency of active sites^29^. By virtue of the definite structure and atomic dispersion of molecular catalysts as well as the remarkable influence of linkage on performance, it is anticipated that heterogenization of the molecular catalyst with an emphasis on linkage will enable the rational design of a target catalyst.

Here, we report the rational design of the hybrid single-atom catalysts (denoted as HSACs) for high-efficiency 2e^−^ ORR electrocatalyst by density-functional theory (DFT) calculations screening on the well-defined molecular metal phthalocyanines (MPc) catalysts. Cobalt phthalocyanine (CoPc) was screened as the candidate and the Co HSACs based on an axial coordination linkage of molecular CoPc with carbon nanotubes (CNT) via pyridine group was calculated. According to theoretical prediction and experimental optimization, we rationally prepared highly efficient Co HSACs that delivers an onset potential of ~0.85 V and H_2_O_2_ selectivity of up to 95%. Furthermore, the Co HSACs catalyst delivers a high current density of 300 cm^−2^ for H_2_O_2_ production in an alkaline flow cell device with natural air diffusion. Unlike the conventional metal-embedded type of SACs, the hybrid of support and molecular catalyst is expected to be a type of HSACs for rationally developing target SACs.

## Results

### Rational screening of MPc catalysts for 2e^−^ ORR by theoretical simulations

**Fig. 1:**
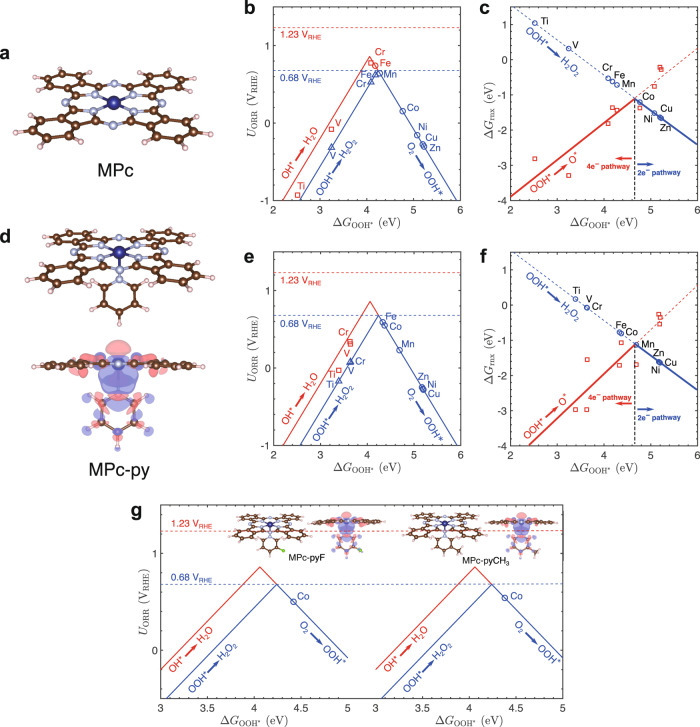
Theoretical predictions of ORR activity and selectivity on various MPc and HSACs based on MPc (M = Ti, V, Cr, Mn, Fe, Co, Ni, Cu, Zn). **a** Typical atomic structure of MPc (color code: dark blue, metal ion; red, O; light blue, N; brown, C; light pink, H). **b** Calculated catalytic activity volcano plot for the 2e^−^ and 4e^−^ reaction pathways of ORR over MPc. **c** Calculated selectivity plot of ORR over MPc. **d** Typical atomic structure of M HSACs (color code: dark blue, metal ion; red, O; light blue, N; brown, C; light pink, H). The isosurface plot shows the charge redistribution upon pyridine ligand linkage to CoPc. The isosurface level is set to 5 × 10^−4^ e/bohr^3^. Red and blue colors represent the electron accumulation and depletion, respectively. **e** Calculated catalytic activity volcano plot for the 2e^−^ and 4e^−^ reaction pathways of ORR over M HSACs. **f** Calculated selectivity plot of ORR over M HSACs. **g** Calculated catalytic activity volcano plot for the 2e^−^ and 4e^−^ reaction pathways of ORR over Co HSACs-F and Co HSACs-CH_3_. The atomic structures of typical Co HSACs-F and Co HSACs-CH_3_ are shown in the insets (color code: dark blue, metal ion; red, O; light blue, N; brown, C; light pink, H; green, F). The isosurface plot shows the charge redistribution upon py-F and py-CH_3_ linkage to CoPc. The isosurface level is set to 5 × 10^−4^ e/bohr^3^. Red and blue colors represent the electron accumulation and depletion, respectively. Source data are provided as a Source Data file.

The DFT calculations were first performed to screen the candidates for 2e^−^ ORR among the MPc (M = Ti, V, Cr, Mn, Fe, Co, Ni, Cu, Zn) molecular catalysts. The adsorption energies of the ORR intermediates (OH^*^, O^*^, and OOH^*^) on the central metal sites are calculated based on the typical molecular structures of MPc (Fig. 1a). Different spin states of the metal ions are considered and the calculated spin-dependent adsorption energies are shown in Supplementary Table 1. To analyze their ORR activity and selectivity, the lowest free energies of adsorption of ORR intermediates on various MPc are adopted (Supplementary Fig. 1a). We further constructed a calculated catalytic activity volcano plot for 2e^−^ (blue) and 4e^−^ (red) reaction pathways of ORR (Fig. 1b)^30^, respectively. The free energy diagrams for ORR on MPc are also plotted (Supplementary Fig. 2).

As shown in Fig. 1b, the binding energies of OOH^*^ for the H_2_O_2_ production are either too strong for VPc, TiPc or too weak for NiPc, CuPc, and ZnPc, resulting in inferior ORR activities compared with those on Mn, Fe, Cr or Co-based MPc. Based on the predictions of ORR activity, we compared the free energy changes of the two competing reaction steps (Supplementary Fig. 2), i.e., the OOH* reduction to H_2_O_2_ and the OOH* reduction to O* and H_2_O, to determine the ORR selectivity for H_2_O_2_. The free energy changes of the two reaction steps as a function of $\triangle {{{\mbox{G}}}}_{{{\mbox{OOH}}}*}$ show an opposite trend with $\triangle {{{\mbox{G}}}}_{{{\mbox{OOH}}}*}$ and intersect at ~4.6 eV (Fig. 1c). It is found that the Co, Ni, Cu, or Zn-based MPc molecules are favorable for H_2_O_2_ production, while the Mn, Fe, Cr, V or Ti-based MPc molecules should be excluded due to their propensity for O–OH bond splitting to produce H_2_O. By comprehensive consideration of both the ORR activity and selectivity for H_2_O_2_ production, the CoPc is thus rationally screened as a promising candidate for 2e^−^ ORR.

In light of the theoretically predicted trend of the 2e^−^ ORR performance, experimental examinations were conducted on the typical late transition-metal-based MPc catalysts. The ORR performance of the above typical samples was evaluated with a catalyst loading of 30 μg cm^−2^ in O_2_-saturated 0.1 M KOH using the rotating ring-disk electrode (RRDE) technique (Supplementary Fig. 3). As displayed in Supplementary Fig. 4, only CoPc catalyst delivers both high activity and selectivity. Specifically, the CoPc catalyst exhibits an onset potential (*E*_onset_) of ~0.8 V (for −0.1 mA cm^−2^) and ~80% H_2_O_2_ selectivity in the wide potential range. Comparatively, the MnPc and FePc show high activity but very poor H_2_O_2_ selectivity (<30% for MnPc, <5% for FePc), whereas the NiPc, CuPc, and ZnPc catalysts exhibit higher H_2_O_2_ selectivity (>80%) but lower activity (*E*_onset_ < 0.7 V). The high performance of CoPc for H_2_O_2_ production is further reflected by the H_2_O_2_ partial current density (*j*_H2O2_), where the *j*_H2O2_ on CoPc is approximately 10 times higher than all other samples at 0.7 V (Supplementary Fig. 5). It is worth noting that the electrochemically active surface area (ECSA) normalized H_2_O_2_ generation activity (Supplementary Figs. 6–8, Supplementary Table 2) is dramatically higher on CoPc with respect to other comparisons, confirming its superior intrinsic activity for 2e^−^ ORR. Owing to the superior performance for 2e^−^ ORR, we mainly focus on CoPc in the following discussion unless otherwise stated.

### Rational design via heterogenization of CoPc molecular catalyst

As far as the CoPc molecule itself is concerned, there are several shortcomings that restrict its activity, selectivity, and stability. First of all, the molecular catalyst is featured with difficult separation, low conductivity, and easy aggregation^31^, which could be addressed by heterogenization of the homogeneous molecule with conducting support. Secondly, the CoPc molecule possesses a typical plane-symmetric structure as well as symmetrical charge distribution in the Co–N_4_ matrix, largely inhibiting the adsorption and activation of the O_2_ molecules. Thirdly, it is easy to see from Fig. 1b that the CoPc locates at the right-hand side of the volcano plot, away from the optimal point for the 2e^−^ ORR. Thus, continuous optimization on the adsorption energy of the OOH* intermediate (i.e., $\triangle {{{\mbox{G}}}}_{{{\mbox{OOH}}}*}$) into a thermoneutral equilibrium potential is highly desirable based on the Sabatier principle^32^. Based on the above analysis, it renders us to make consideration on how to break its structural symmetry and modify its local electronic structure as well as $\triangle {{{\mbox{G}}}}_{{{\mbox{OOH}}}*}$ via the heterogenized strategy.

Inspired by the unique structure of natural ORR metal enzyme that contains a five-coordinated configuration with an axial coordination^33,34^, we further employ DFT calculations to evaluate the possibility of modulating the electronic structure of Co by axial coordination linkage between CoPc and CNT support via pyridine group to generate the Co HSACs (Fig. 1d–f, Supplementary Figs. 1b, 9, and Supplementary Table 3). Pyridine group (py) is chosen because of its robust grafting with CNT by the covalent interaction and facile linkage with Co ion through coordination bond. In addition, the size of pyridine is small which enables immediate charge transfer between the support and active sites. It is reasonable to expect that the additional coordination of CoPc with pyridine group will form the Co–N_5_ structure in the Co HSACs and break the charge symmetry of the pristine Co–N_4_ moiety in CoPc. Notably, the Co–N_5_ structure in the Co HSACs does modulate the $\triangle {{{\mbox{G}}}}_{{{\mbox{OOH}}}*}$ of Co active center for higher ORR activity and maintain high selectivity for 2e^−^ process, as indicated by the volcano plot and the selectivity plot (Fig. 1e, f). $\triangle$G_OOH*_ on Co HSACs (Fig. 1e) is greatly decreased by about 0.4 eV comparing to that on CoPc (Fig. 1b). The charge redistribution plot in Fig. 1d exhibits a remarkable electron accumulation upon pyridine linkage over the Co active center, and the accumulated electrons should be occupying the Co 3d_z_^2^ orbital as suggested by the shape of the orbital lobe. We attribute the decrease of $\triangle$G_OOH*_ to this electron accumulation upon pyridine introduction, which enhances the interactions between Co 3d_z_^2^ orbital and p orbital in OOH. The origin of the electron accumulation over the Co active center is due to the coordinate bond formed between CoPc and pyridinic N. The latter possesses a lone pair of electrons that can repel the electron occupying Co 3d_z_^2^ orbital in CoPc upon the coordinate bond formation, thus induces the electron accumulation over the Co active center. Furthermore, we attempt to finely modulate the local electronic structure of the Co center by attaching the electron-withdrawing fluorine group or electron-donating methyl group on pyridine linkage to form Co HSACs-F and Co HSACs-CH_3_ (Fig. 1g)^21,35^, respectively. The calculated DFT results can be accessed in Fig. 1g, Supplementary Figs. 10–13, and Supplementary Tables 4, 5. The volcano plots in Fig. 1g demonstrate that F and CH_3_ groups could regulate the electronic structure, but the modification effect is minor because DFT calculations usually take approximate approach.

**Fig. 2:**
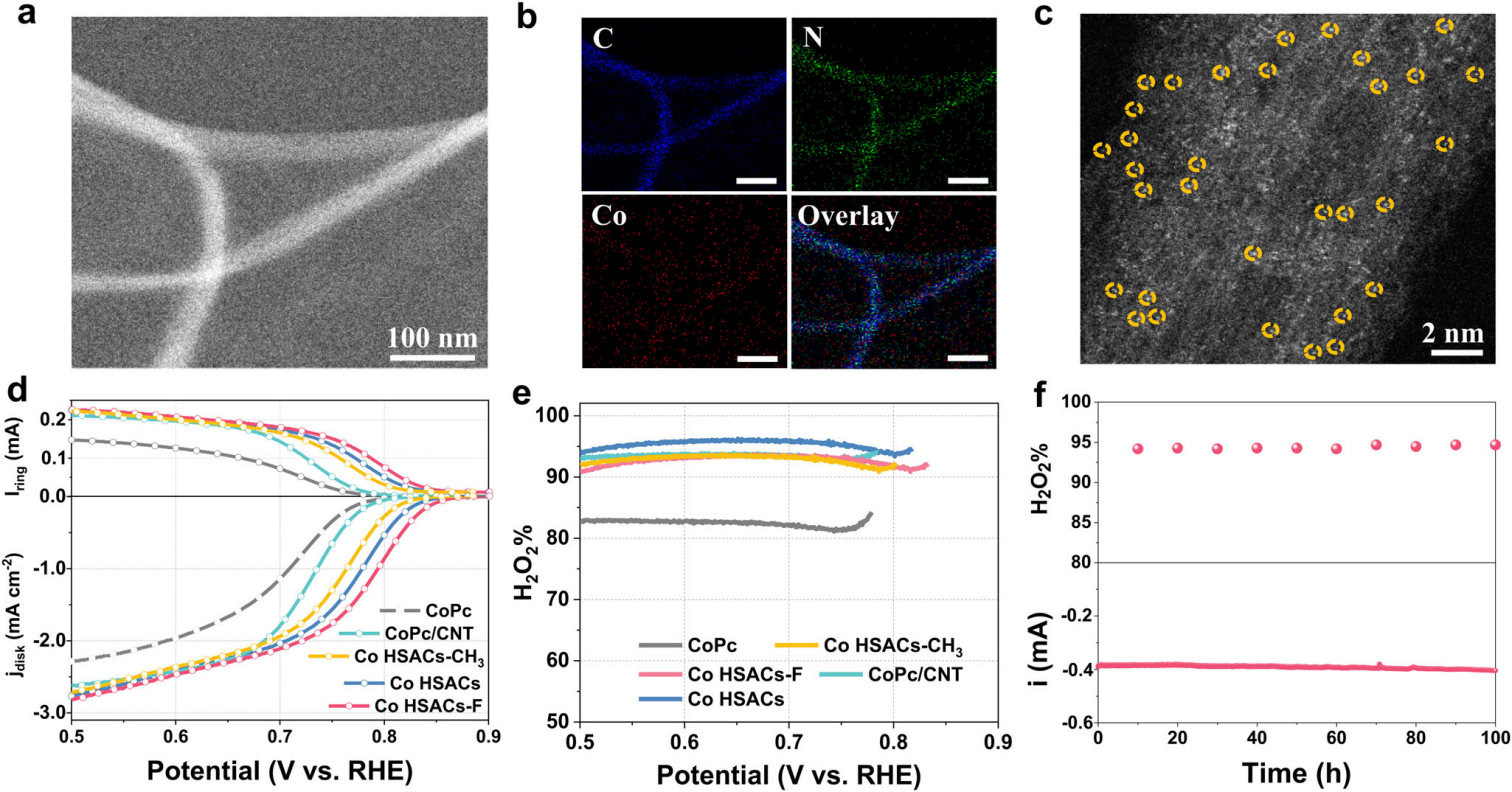
Structural and performance characterization of Co HSACs. **a**, **b** Aberration-corrected HAADF-STEM image of Co HSACs (**a**) and corresponding EDX mapping of C, N, Co, and overlay (**b**). Scale bars, 100 nm (**b**). **c** Aberration-corrected atomic-resolution HAADF-STEM image of Co HSACs. The bright spots of Co atoms are marked with red circles. **d** Comparison of ORR performance at 1600 rpm and the simultaneous H_2_O_2_ detection current densities at the ring electrode for Co HSACs, Co HSACs-F, Co HSACs-CH_3_, CoPc/CNT, and CoPc in 0.1 M KOH. **e** The calculated H_2_O_2_ selectivity as a function of the applied potential. **f** Stability test of Co HSACs catalyst under 0.5 V bias vs. RHE in O_2_-saturated 0.1 M KOH, rotation speed 500 rpm. Source data are provided as a Source Data file.

On the basis of the DFT predictions, we experimentally prepared Co HSACs by first covalent decoration of pyridine group on the CNT^36^ and then coordination with the CoPc molecule (see details in Methods and Supplementary Fig. 14). A variety of characterizations shown in Supplementary Figs. 15–19 and Supplementary Table 6 confirm the successful coordination linkage for the heterogenization. The content of CoPc in the Co HSACs was optimized to be ca. 0.7 at% (Supplementary Fig. 20). Co HSACs-F and Co HSACs-CH_3_ were prepared by applying the same protocol as that of the Co HSACs (see details in Methods). For comparison, the mechanical mixture of CoPc and CNT (denoted as CoPc/CNT) was also synthesized. High-angle annular dark-field scanning transmission electron microscopy (HAADF-STEM) images and the corresponding STEM elemental mapping exhibit the homogeneous distribution of Co and N throughout the carbon nanotubes (Fig. 2a, b). The single Co atoms are readily visible as bright dots on the surface of CNT by atomic-resolution HAADF-STEM image (Fig. 2c), where the atoms are observed with uniform distribution^37,38^.

In comparison to pristine CoPc or CoPc/CNT mixture, the Co HSACs catalyst exhibits remarkably promoted ORR activity and selectivity to H_2_O_2_ (Fig. 2d, e). Moreover, the well-defined structure of our heterogenized molecular Co HSACs catalyst allows tuning of ORR catalytic property with atomic precision, which is difficult to achieve with heterogeneous electrocatalysts due to the inhomogeneity of active site geometries and local electronic structures^39^. For instance, the ORR activity of Co HSACs can be finely tuned by appending electron-withdrawing -F group or electron-donating –CH_3_ group on the pyridine linkage. As given by Bader charge analysis, the Bader charge of the N atoms are 2.74e, 2.73e, and 2.86e in pyridine, pyridine-F, and pyridine-CH_3_, respectively, suggesting the electron drawing and donating capabilities of F and CH_3_ ligands. As a result, the ORR activities on Co HSACs-F or Co HSACs-CH_3_ are further increased or decreased, respectively, as indicated by the linear sweep voltammetry (LSV) curves (Fig. 2d, e). Notably, the above experimental results are in good agreement with the theoretical calculation results. The H_2_O_2_ selectivity on the Co HSACs exceeds 95% in a wide potential range of 0.75–0.5 V (Fig. 2e), which is further confirmed by the electron-transfer number (n) calculated from the Koutecky-Levich (K-L) plots (Supplementary Fig. 21). The Tafel slopes of typical samples indicate the faster reaction kinetics of Co HSACs (Supplementary Fig. 22). At the applied potential of 0.7 V, the Co HSACs delivers a turnover frequency (TOF) of 0.89 s^−1^, about 7.4 times of CoPc with the TOF of 0.12 s^−1^ (see details in Method, Supplementary Table 7). It is worth noting that the Co HSACs delivers an onset potential ~0.85 V (for −0.1 mA cm^−2^) beyond the thermodynamic limit (0.76 V in 0.1 M KOH, pH = 13), as should similarly result from a Nernst-related potential shift caused by the generated H_2_O_2_ near the double-layer of catalyst (Supplementary Note 1)^40,41^. The stability of Co HSACs was tested at 0.5 V using an H-type electrochemical cell. After a prolonged operation of 100 h, the current was nearly retained, demonstrating its long-term stability (Fig. 2f). To our knowledge, the performance of Co HSACs surpasses the state-of-the-art catalysts for 2e^−^ ORR with the most positive onset potential and comparable high H_2_O_2_ selectivity (Supplementary Table 7). Interestingly, we find the other M HSACs (M = Mn, Ni, Cu, Zn) also deliver enhanced 2e^−^ ORR activity with comparable selectivity, suggesting the applicability of the design strategy (Supplementary Figs. 23, 24).

**Fig. 3:**
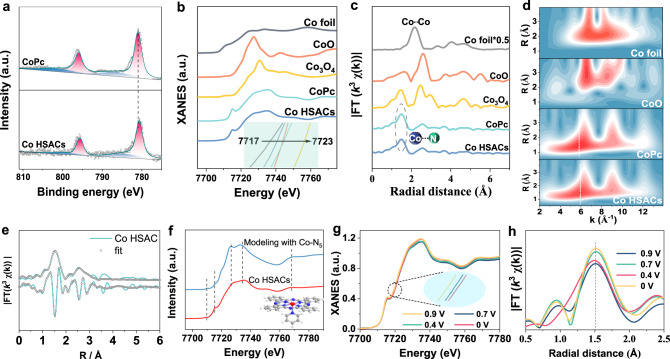
Electronic states of Co atom in Co HSACs and operando XAS characterization. **a** High-resolution XPS spectra of Co 2*p* for Co HSACs. **b** The normalized K-edge XANES spectra. **c** EXAFS spectra (without phase correction) of Co HSACs, CoPc, and reference samples. **d** Wavelet transform (WT) for the k^3^-weighted EXAFS signals. **e** FT-EXAFS fitting spectra for Co HSACs in R space. **f** Comparison between the Co K-edge XANES experimental spectra and the theoretical spectra calculated with the depicted structure (inset) for HSACs. **g** Normalized operando Co K-edge XANES spectra for Co HSACs at various biases (applied voltage vs RHE) in 0.1 M KOH solution at room temperature in 1 atm of O_2_. The inset shows the enlarged Co K-edge XANES spectra. **h** Fourier transform magnitudes of EXAFS spectra (without phase correction) of Co HSACs under various biases. Source data are provided as a Source Data file.

The modulation of the axial coordination linkage on the electronic structure of Co active center was confirmed by the X-ray photoelectron spectroscopy (XPS) (Fig. 3a) and X-ray absorption spectroscopy (XAS) (Fig. 3b–d). Compared with the pristine CoPc, the lower valence state of Co on the Co HSACs can be deduced from its lower binding energy of the Co2*p*_3/2_ XPS peak and negatively shifted Co absorption edge of X-ray absorption near-edge spectroscopy (XANES), respectively. This should be due to the electron-donating property of the pyridine functional group in the Co HSACs, leading to increased electron density around the Co atoms^42^. The extended X-ray absorption fine structure (EXAFS) spectra in Fig. 3c display only one peak at 1.48 Å, corresponding to the primary coordination shell of Co. In addition, no characteristic Co–Co peaks (typically at 2.16 Å) associated with the aggregated Co atoms are found in Co HSACs, consistent with the HAADF-STEM results (Fig. 2c).

The existence of axial coordination linkage in the Co HSACs is supported by EXAFS spectra (Fig. 3c-f, Supplementary Figs. 25–27, and Supplementary Table 8). Specifically, the Co–N coordination number (C.N.) is estimated to be ca. 5 with an average bond length of 1.92 Å for Co HSACs, in contrast to the C.N. of ca. 4 for the pristine CoPc (Fig. 3e, Supplementary Table 8). It suggests that the Co single atom in Co HSACs is coordinated with five N atoms. Considering that the pristine CoPc only contains Co–N_4_ structure, it is reasonable to deduce that the additionally coordinated N should originate from the pyridine functional group^43^. The wavelet transform (WT) plot (Fig. 3d) of Co HSACs shows the WT maximum at ~6 Å^−1^, corresponding to the Co–N bonding by comparing with Co foil, CoO, and CoPc. No intensity maximum corresponding to Co–Co can be observed, which agrees with the EXAFS results (Fig. 3c). Furthermore, in order to verify the geometric structure of Co HSACS, we performed XANES simulation calculations. It can be found that the calculated spectra for five coordinated Co–N_5_ moiety with axial linkage can reproduce well the experimental spectra of the sample (Fig. 3f), and the shoulder peak at ~7717 eV is weakened compared with CoPc sample (Supplementary Fig. 28). Consequently, the combination of XPS, EXAFS fitting, and XANES simulation together identified the five coordination of Co site with one axial linkage.

To get insight into the adsorption and activation of O_2_ on the Co HSACs, Co K-edge XANES and EXAFS spectra were performed under operando conditions (Supplementary Fig. 29)^38^. Figure 3g, h displays the normalized Co K-edge XANES spectra and the corresponding Fourier transforms of the EXAFS spectra, respectively. As shown in Fig. 3g and the inset, Co K-edge moves to a higher energy position with the increasing cathodic bias from open-circuit potential (OCP) to 0.4 V, suggesting the increasing valence states of Co atoms. This probably results from the delocalization of the unpaired electron in the 3d*x*^2^–*y*^2^ orbital and spontaneous charge transfer from Co (II) to the oxygen 2p orbital in O_2_ to form an *OOH intermediate on the Co site^37,44^. It is worth noting that the Co valence state increases from 0.9 V to 0.7 V and reaches the plateau at 0.4 V, owing to the mass-transfer controlled diffusion of O_2_ at 0.4 V that resulted in the limiting current of 2e^−^ ORR, as indicated in Fig. 2d. Furthermore, the possible coordination of *OOH intermediate with the Co site is reflected by the slight increase of peak intensity at approximately 1.50 Å in the Fourier transform of the EXAFS spectrum (Fig. 3h), leading to a varied coordination environment of the Co site. However, the obvious shift in bond length is not observed due to the similar bond length of Co–N and Co–O bond.

### Air-breathing flow cell for high-rate H_2_O_2_ production

**Fig. 4:**
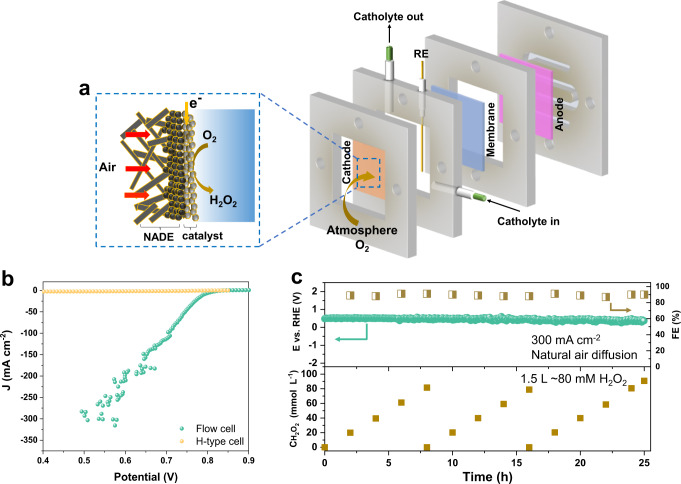
Flow cell performance of catalysts. **a** Schematic illustration of the air-breathing flow cell configuration with natural air diffusion electrode. **b** Comparison of polarization curves of Co HSACs in the flow cell and H-type cell with natural air. Electrolyte: 0.5 M KOH. **c** Stability test of the Co HSACs under 300 mA cm^−2^ in 0.5 M KOH for practically producing 80 mM H_2_O_2_ solution. The volume of 0.5 M KOH solution is 500 mL. The electrolyte feeding rate was fixed at 2 mL min^−1^, and the oxygen is fed by the natural air diffusion. EDTA was added to the catholyte for the flow cell. The electrolyte was sampled every 2 h to determine the Faradaic efficiency and the H_2_O_2_ concentration, and the electrolyte was refreshed at the same time interval. Source data are provided as a Source Data file.

Encouraged by the extraordinarily high activity and selectivity to produce H_2_O_2_ and good stability on the Co HSACs during the RRDE tests, we sought to apply a three-phase flow cell reactor to evaluate the potential for high-rate production of H_2_O_2_ on Co HSACs. For industrial applications, aeration energy consumption accounts for a proportion of the total energy consumption which cannot be neglected^45^. To improve the energy utilization efficiency, we applied a natural air diffusion electrode (NADE) to enhance the oxygen mass transfer, which allows air to naturally diffuse to the ORR interface without additional pure O_2_ aeration (see Methods for details, Supplementary Fig. 30)^46^. As depicted in Fig. 4a, the catalysts were coated onto the NADE and positioned against the electrolyte, while the back of the NADE is exposed to the air for natural air diffusion to the triphase interface. The current-voltage (I-V) curves were recorded at a fixed flow rate of electrolyte (2 mL min^−1^) with natural air diffusion in 0.5 M KOH solution. Strikingly, the Co HSACs delivers an ORR activity two orders of magnitude higher than that in the H-cell (Fig. 4b, Supplementary Fig. 31), suggesting the enormous contribution from the enhanced O_2_ diffusion. The current density quickly increases upon the onset potential and reaches 300 mA cm^−2^ at a positive potential of ~0.55 V. Moreover, the Co HSACs can retain high Faradaic efficiency of over 90% at 300 mA cm^−2^ for at least 25 h without obvious degradation in both activity and selectivity (Fig. 4c). Meanwhile, the H_2_O_2_ concentration continuously increased to ~80 mM in about 8 h in 500 mL KOH solution, which can be stably operated for 3 cycles and produced 1.5 L of 80 mM H_2_O_2_ solution. Its robustness can be also supported by the marginal change in the morphology, electronic structure, and coordination environment of catalyst after the durable ORR test under the high current density (Supplementary Figs. 32–35, Supplementary Table 9).

## Discussion

In conclusion, we introduce an alternative approach to rationally develop HSACs for high-efficiency 2e^−^ ORR by DFT prediction and experimental confirmation. Distinct from the conventional metal-embedded SACs, our HSACs, composed of single atom dispersed molecule, covalent linkage, and support, own unambiguous structure of active site for definite correlation with its catalytic performance. Furthermore, they allow fine modulation of the electronic structure for optimization of performance, which collectively enables the rational design of the target catalyst. In this work, we provide a clear model illustration on the rational design and fabrication of HSACs toward high-efficiency 2e^−^ ORR, where the DFT calculation plays an important role in screening the MPc molecular candidate and predicting the modulation effect of axial coordination linkage on the electronic structure by heterogenization. Our experimental results well verify the theoretically predicted trend, and finally develop a highly efficient Co HSACs that exhibits up to 95% 2e^−^ ORR selectivity and 0.85 V onset potential in an H-cell, and give high-rate production of H_2_O_2_ at 300 mA cm^−2^ in an air-breathing flow cell. Strikingly, the as-developed HSACs exhibit comparable ORR stability as conventional heterogeneous metal-embedded SACs. The HSACs combine the merits of conventional SACs and the well-defined structure of atomic active sites, which can be expected as a new generation of SACs for the understanding of underlying reaction mechanism and rational design of target catalysts.

## Methods

### Chemicals

Sodium nitrite (NaNO_2_, ≥99.99%), 4-aminopyridine (98%), Dimethyl formamide (DMF), and tetrahydrofuran (THF) were purchased from Aladdin. Cobalt phthalocyanine (Co(II)Pc, 97%), iron phthalocyanine (Fe(II)Pc, 98%), copper phthalocyanine (Cu(II)Pc, 90%), zinc phthalocyanine (Zn(II)Pc, 95%), nickel phthalocyanine (Ni(II)Pc, 95%), and manganese phthalocyanine (Mn(II)Pc, 97%) were purchased from Alfa-Aesar. Cerium sulfate (Ce(SO_4_)_2_) was purchased from Sigma-Aldrich. Single-walled carbon nanotubes (CNT) were purchased from Timesnano. Nafion ionmer (5 wt% in isopropanol/H_2_O, Dupont) was diluted to 0.25 wt% with EtOH. All aqueous solutions were prepared using deionized water (18.2 MΩ cm) obtained from an ultra-pure purification system.

### Purification of CNT

Pristine CNT was purified by heating in air at 400 °C and then underwent acid washing in 6 M HCl at 80 °C for 12 h. The purified CNT was washed with a copious amount of water until pH neutral and dried in vacuum. Afterward, the CNT was annealed at 900 °C in an argon atmosphere to remove residual oxygen-containing functional groups and any adsorbed gases or solvents.

### Synthesis of pyridine-functionalized CNT (CNT-py)

An icy-cold solution (0 °C) of NaNO_2_ (1.41 g) dissolved in 2 mL of water was added dropwise to 14.4 mL of 5 M HCl dissolved with 4-aminopyridine (1.90 g) cooled to 0 °C, and then kept in an ice bath. The resulting yellow solution was stirred for 30 min and maintained at 0 °C. Subsequently, an icy-cold solution (0 °C) of purified CNT (28.8 mg) well-dispersed in DMF by sonication (2 h) was added dropwise. The reaction mixture was held at 0 °C by ice bath and stirred for 4 h, followed by natural warm to room temperature and stirred for a further 20 h. The grafted CNT was collected by centrifugation, redispersed in 2 M HCl (100 mL), filtered, and washed with a copious amount of water until pH neutral. The resulting solid material was dispersed in 2 M NaOH (100 mL) and stirred overnight to ensure deprotonation of the pyridinium salt to pyridine. Afterward, the functionalized CNT was filtered and thoroughly washed with water until pH neutral, redispersed and centrifugated with THF, acetone, and ethanol, respectively, and dried in vacuum to afford CNT-py.

### Synthesis of Co HSACs

The Co HSACs were prepared by refluxing the mixture of the as-prepared CNT-py and CoPc in THF under the protection of argon gas for 1 h. The loading amount of CoPc in the HSACs was selected according to the content of pyridine group in the CNT. Since the N content which corresponds to the pyridinic group on the CNT is 1.43at% by XPS (Supplementary Table 6), we selected three different CoPc loading amounts (0.7at%, 1.4at%, and 2.1at%) to link with CNT-py to determine the optimal loading content. The resulting 2e^-^ ORR examination shows that the Co HSACs with Co content of 0.7at% delivers similar performance. To achieve higher turnover frequency, we thus select 0.7at% for the discussion.

### Synthesis of Co HSACs-F and Co HSACs-CH_3_

The preparation of Co HSACs-F and Co HSACs-CH_3_ is similar to that of the Co HSACs, except that the 4-aminopyridine was changed to 4-amino-3-fluoropyridine and 4-amino-3-methylpyridine during the synthesis of functionalized CNT.

### Synthesis of CoPc/CNT mixture

CoPc/CNT mixture was obtained by the direct physical mixture of CoPc and CNT in the mortar.

### Structural and morphological characterization

The morphologies of the electrodes were performed by a Quanta 200 FEG scanning electron microscope (SEM) equipped with an energy-dispersive spectrometer (accelerating voltage of 20 kV). TEM micrographs were taken on a FEI Tecnai G^2^ F30 transmission electron microscope. High-angle annular dark-field scanning transmission electron microscopy (HAADF-STEM) images were obtained on the JEOL JEMARM300F STEM with a guaranteed resolution of 0.063 nm, as well as a dual SDD energy-dispersive spectroscopy (EDS) detector. Samples were dispersed by ultrasonication in ethanol, and resulting dropped onto Cu mesh with carbon microgrids. Thermo Fisher Esca Lab 250 Xi spectrometer was used to perform the XPS measurements with a monochromatic Al K α source (Ephoton = 1486.6 eV) with a 6-mA. All XPS spectra were calibrated with respect to adventitious carbon C1*s* peak shifted to 284.6 eV. Raman spectra acquisition was performed using a Renishaw InVia Raman microscope and 532 nm excitation laser.

### Thermo gravimetric analysis (TGA)

TGA data were recorded on ca. 2 mg of the sample using PE Diamond TG/DTA equipment. Data were recorded in flowing N_2_ (100 mL min^−1^) with a heating rate of 10 °C min^−1^ to 900 °C after being held at 120 °C for 30 min to remove any residual solvent.

### XAFS measurement

The X-ray absorption fine structure (XAFS) spectra at Co K-edge were collected at 1W1B beamline of Beijing Synchrotron Radiation Facility (BSRF) and BL14W1 beamline of Shanghai Synchrotron Radiation Facility (SSRF). The data were collected in fluorescence mode using a Lytle detector and the corresponding reference samples were collected in transmission mode.

In situ XAFS measurement was conducted with a homemade H-type cell. This cell contains three electrodes with a carbon foil coated by catalyst as the working electrode, Pt mesh as the counter electrode, and Ag/AgCl as the reference electrode, respectively. The catalyst was drop-cast on the carbon foil and allowed to dry. A small window was cut out on the cathode side and sealed with Kapton film to allow fluorescence signals to pass from the electrode to the detector. The electrolyte was obtained by bubbling O_2_ into the 0.1 M KOH aqueous solution before testing. The XAFS spectra were collected under open-circuit potential and applied potentials.

The acquired XAFS data were processed according to the standard procedures using the ATHENA module of Demeter software packages. To obtain the quantitative structural parameters around central atoms, least-squares curve parameter fitting was performed using the ARTEMIS module of Demeter software packages.

The following EXAFS equation was used:

1 $$\chi (k)=\mathop{\sum}\limits_{j}\frac{{N}_{j}{S}_{0}^{2}{F}_{j}(k)}{k{R}_{j}^{2}}\cdot {{{{{\rm{exp}}}}}}[-2{k}^{2}{\sigma }_{j}^{2}]\cdot {{{{{\rm{exp}}}}}}\left[\frac{-2{R}_{j}}{\lambda (k)}\right]\cdot {{{{{\rm{sin}}}}}}[2k{R}_{j}+{\phi }_{j}(k)]$$

### XANES simulations

The simulations were performed using the FDMNES program, which is based on the Finite Difference Method (FDM) to solve the Schrödinger equation and uses the Green formalism (multiple scattering) on a muffin-tin potential^47^. During the XANES simulations of Co HSACs sample, the models for simulations were constructed according to the EXAFS curve fittings. The final states were calculated in a Co-atom-centered sphere with a fixed radius, with the atoms inside the sphere considered in the calculation. To achieve a balance between the good calculation and time spending, a radius of 7 Å was selected for the sphere.

### Sample preparation for the electrochemical test

The catalyst ink was prepared by dispersing the electrocatalysts in 0.25 wt% Nafion-ethanol to achieve a catalyst concentration of 2.5 mg ml^−1^. After sonication for 60 min, catalyst ink was drop-dried onto the glassy-carbon disc of the RRDE or RDE electrode to get a loading amount of 0.03 mg cm^−2^. The sample preparation of MPc catalysts without CNT was the same to the above protocol, but the MPc was loaded onto the glassy carbon electrode without the support.

### Electrochemical characterization

All electrochemical experiments were conducted with a CHI760E electrochemical working station. The saturated calomel electrode (SCE), carbon rod, and glassy carbon electrode loaded with electrocatalyst were used as the reference electrode, counter electrode, and the working electrode, respectively. Electrochemical measurements were implemented in an O_2_-saturated 0.1 M KOH aqueous solution at temperature range of 22–25 °C controlled by an air conditioner. The rotating speed of the electrode was set as 1600 rpm unless otherwise stated and the ring potential was constant at 1.5 V vs_._ RHE. Linear sweep voltammetry (LSV) measurements were conducted at a scan rate of 10 mV s^−1^. The measured potentials using a three-electrode setup of RDE and RRDE have no *iR*-compensation, the potentials in flow cell were manually 100% *iR*-compensated. The error of experimental data can be obtained by calculating the mean and standard deviation.

All the electrode potentials were converted by referring to the reversible hydrogen electrode (RHE) based on Eq. (2):

2 $${E}_{({{{{{\rm{RHE}}}}}})}={E}_{({{{{{\rm{SCE}}}}}})}+pH\times 0.059+{E}_{({{{{{\rm{SCE}}}}}})}^{o}$$

where *E*^o^_(SCE)_ (saturated KCl) = 0.242 V (25 °C).

### Rotating disk electrode (RDE) test

For the RDE measurements, the electron-transfer number per oxygen molecule involved was calculated based on the Koutecky–Levich (K–L) equation (Eq. (3)), where *J* is the measured current, *J*_*k*_ is the kinetic-limiting current, and *ω* is the electrode rotation rate. By changing the electrode rotation rate, we can get a series of current values (*J*). A linear plot could then be obtained by plotting *J vs ω*. The electron-transfer number (n) per oxygen molecule involved could be calculated from the slope (B) of the linear plot (Eq. (4)), where n is the overall number of transferred electrons in the ORR process, F is the Faradaic constant (96485 C mol^−1^), C_O2_ is the oxygen concentration (solubility) in 0.1 M KOH (1.2 × 10^−6 ^mol cm^−3^), D_O2_ is the oxygen diffusion coefficient in 0.1 M KOH (1.9 × 10^−5^ cm^2^ s^−1^) and *ν* is the kinematic viscosity of 0.1 M KOH (0.01 cm^2^ s^−1^).

3 $${J}^{-1}={J}_{L}^{-1}+{J}_{K}^{-1}={B}^{-1}{\omega }^{-1/2}+{J}_{K}^{-1}$$

4 $$B=0.62nF{C}_{{O}_{2}}{D}_{{O}_{2}}^{2/3}{v}^{-1/6}$$

For an ideal catalyst, the expected shape of the voltammetric profile should have a sigmoidal shape, reaching a maximum value, which is named as limiting diffusion current. This current value is obtained when the electrode kinetics is very fast and the reaction rate is controlled by the diffusion of the reactants to the surface of the electrode. This diffusion-limited current can be easily derived using the Levich equation:

5 $${i}_{{{{{\mathrm{lim}}}}}}=-0.62nAF{D}^{2/3}{v}^{-1/6}{c}_{b}{\omega }^{1/2}$$

where *n* is the number of electrons exchanged in the process, *A* is the geometrical area of the electrode, *D* is the diffusion coefficient, *v* is the kinematic viscosity, *c*_b_ is the bulk concentration of oxygen, and *ω* is the rotation rate. Using the reported values *D* and *c*_b_ for oxygen and *v*, and the geometrical area of the electrode (0.197 cm^2^), a current value of 0.430 mA is obtained when H_2_O_2_ is formed (*n* = 2) or 0.865 mA for water (*n* = 4) for 1600 rpm. These values are equivalent to current density values per geometrical area of 2.19 and 4.39 cm^−2^, respectively.

### Rotating ring-disk electrode (RRDE) test

The rotating-ring-disk electrode (RRDE) assembly consisted of a glassy-carbon rotation disk electrode (disk area 0.2475 cm^2^) and a Pt-ring (ring area 0.1866 cm^2^). The collection efficiency was determined using K_3_Fe[CN]_6_/K_2_Fe[CN]_6_ as the redox couple. The plots of collection efficiency were collected at −0.2 V and −0.4 V at different rotation rates. The disk electrode was scanned cathodically at a rate of 10 mV s^−1^ and the ring potential was constant at 1.5 V vs. RHE. The HO_2_^-^% and the n were determined by the following Eqs. (6) and (7):

6 $$H{O}_{2}^{-}\%= 200\times \frac{{{{{{{\rm{I}}}}}}}_{r}/N}{{I}_{d}+{I}_{r}/N}$$

7 $$n=4\times \frac{{I}_{d}}{{I}_{d}+{I}_{r}/N}$$

where *I*_d_ is disk current, *I*_r_ is ring current and *N* is the current collection efficiency (*N*) of the Pt ring. *N* was determined to be 0.38 from the reaction of reduction of K_3_Fe[CN]_6_/K_2_Fe[CN]_6_.

The Tafel slopes were calculated by the sweep point method according to the Tafel equation (Eq. (8)):

8 $$\eta=b\,\log (j/{j}_{o})$$

where *η* is overpotential, b is Tafel slope, *j* is current density, and *j*_*0*_ is the exchange current density. Firstly, we achieved the source current by maintaining the potential at each point for a period of 20 s to ensure steady-state conditions. Based on this, we obtained the Tafel slope for each sample from the *J*–*V* plots using a linear fit applied to points in the Tafel region. The ECSA was determined by measuring the capacitive current associated with double-layer charging from the scan-rate dependence of CVs. For this, the potential window of CVs was −0.1 to 0.1 V versus SCE with Ar-saturated condition. The scan rates were 10, 20, 50, 100, 200, 400, and 800 mV s^−1^. The double-layer capacitance (*C*_dl_) was estimated by plotting the Δ*j* = (*j*_*a*_ − *j*_*c*_)/2 at 0 V versus SCE against the scan rate. The ECSA values were calculated from the measured double-layer capacitance divided by the specific capacitance of an atomically smooth material (*C*_s_, ~20 μF cm^−2^): ECSA = *C*_*dl*_ ÷ *C*_s_ × *S*, where *S* is the actual surface area of the electrode.

### H_2_O_2_ concentration measurement

The concentration of H_2_O_2_ was measured by spectroscopic titration using the cerium sulfate (Ce(SO_4_)_2_) as the reagent. The reaction mechanism is that a yellow solution of Ce^4+^ could be reduced by H_2_O_2_ to colorless Ce^3+^ (Eq. (9)). Thus, the concentration of Ce^4+^ before and after the reaction can be measured by ultraviolet-visible spectroscopy. The absorbance at 316 nm was measured (*A*_s_) on a spectrophotometer (Shimadzu UV-2600).

9 $$2{{{{{\rm{C}}}}}}{{{{{{\rm{e}}}}}}}^{4+}+{{{{{{\rm{H}}}}}}}_{2}{{{{{{\rm{O}}}}}}}_{2}\to 2{{{{{\rm{C}}}}}}{{{{{{\rm{e}}}}}}}^{3+}+2{{{{{{\rm{H}}}}}}}^{+}+{{{{{{\rm{O}}}}}}}_{2}$$

Therefore, the amount of H_2_O_2_ (*M*) could be determined by the Eq. (10):

10 $$M=1/2\times M(C{e}^{4+})$$

where *M(*Ce^4+^) is the mole of consumed Ce^4+^.

We prepared 1 mM of yellow transparent Ce(SO_4_)_2_ solution by dissolving Ce(SO_4_)_2_ salts in 0.5 M H_2_SO_4_ acid solution. Then, the calibration curve was obtained by adding H_2_O_2_ solutions with a certain concentration to Ce(SO_4_)_2_ solution and measured with ultraviolet-visible spectroscopy. The concentration of H_2_O_2_ in the electrolyte can be derived based on the linear relationship between the signal intensity and Ce^4+^ concentration. The difference in absorbance was determined as follows: Δ*A*_318_ = *A*_B_ – *A*_s_. Based on the above results, the amount of H_2_O_2_ was determined and shown in Supplementary Fig. 36.

### Calculation of turnover frequency (TOF) for the electrochemical H_2_O_2_ production

We used the turnover frequency (TOF) values of catalysts to evaluate the efficiency of 2e^−^ ORR pathway for H_2_O_2_ production. The turnover frequency (TOF) is the total number of molecules transformed into desired product by one active site per time. To this end, the activity of different catalysts was evaluated by comparing their TOF values which consider the differences in the site density of various catalysts. The equation for deriving TOF is listed below.

11 $${{{{{\rm{TOF}}}}}}({{{{{{\rm{s}}}}}}}^{-1})=({{{{{\rm{number}}}}}}\,{{{{{\rm{of}}}}}}\,{{{{{\rm{oxygen}}}}}}\,{{{{{\rm{molecules}}}}}}\,{{{{{\rm{turnover}}}}}})/({{{{{\rm{number}}}}}}\,{{{{{\rm{of}}}}}}\,{{{{{\rm{active}}}}}}\,{{{{{\rm{sites}}}}}})=(j/2F)/n$$

*j* is the current density for H_2_O_2_ production measured from the ring electrode with the collection efficiency of the RRDE setup at a given overpotential, *F* is the Faradaic constant (96485 C mol^−1^), and n is the number of active sites. ((*j*/2 *F*) is the total oxygen turnover in 2e^−^ ORR.

The number of oxygen molecules turnover was calculated as follows:

12 $$	({{{{{\rm{number}}}}}}\,{{{{{\rm{of}}}}}}\,{{{{{\rm{oxygen}}}}}}\,{{{{{\rm{molecules}}}}}}\,{{{{{\rm{turnover}}}}}})=j\left[\frac{{{{{{\rm{mA}}}}}}}{{{{{{\rm{c}}}}}}{{{{{{\rm{m}}}}}}}^{2}}\right]\times \frac{1[\frac{{{{{{\rm{C}}}}}}}{{{{{{\rm{s}}}}}}}]}{1000[{{{{{\rm{mA}}}}}}]} \\ 	 \times \frac{1[{{{{{\rm{mol}}}}}}\cdot {{{{{{\rm{e}}}}}}}^{-}]}{96485[{{{{{\rm{c}}}}}}]}\times \frac{1[{{{{{\rm{mol}}}}}}\cdot {{{{{{\rm{O}}}}}}}_{2}]}{2[{{{{{\rm{mol}}}}}}\cdot {{{{{{\rm{e}}}}}}}^{-}]}\times \left(6.02\times {10}^{23}\left[\frac{{{{{{\rm{atom}}}}}}\cdot {{{{{{\rm{O}}}}}}}_{2}}{{{{{{\rm{mol}}}}}}\cdot {{{{{{\rm{O}}}}}}}_{2}}\right]\right)$$

The number of active sites was calculated as follows:

13 $$({{{{{\rm{number}}}}}}\,{{{{{\rm{of}}}}}}\,{{{{{\rm{active}}}}}}\,{{{{{\rm{sites}}}}}}),n=	 L\left[\frac{{{{{{\rm{mg}}}}}}}{{{{{{\rm{c}}}}}}{{{{{{\rm{m}}}}}}}^{2}}\right]\\ 	 \times R[{{{{{\rm{wt}}}}}} \%]\times \frac{1[{{{{{\rm{mmol}}}}}}]}{W[{{{{{\rm{mg}}}}}}]}\times \left(6.02\times {10}^{20}\left[\frac{{{{{{\rm{atom}}}}}}}{{{{{{\rm{mmol}}}}}}}\right]\right)$$

*L* is the amount of catalyst loaded on the electrode, *R* is the weight fraction, and *W* is the atomic weight of the corresponding element of active sites.

Herein, we consider Co as the main active site, due to the much higher 2e^−^ ORR activity of CoPc and CoPc-py-CNT compared with that of the bare Pc. Therefore, the TOF value at 0.7 V (vs. RHE) is calculated as follows:

$$	({{{{{\rm{number}}}}}}\,{{{{{\rm{of}}}}}}\,{{{{{\rm{oxygen}}}}}}\,{{{{{\rm{molecules}}}}}}\,{{{{{\rm{turnover}}}}}})=2.0 \left [\frac{{{{{{\rm{mA}}}}}}}{{{{{{\rm{c}}}}}}{{{{{{\rm{m}}}}}}}^{2}} \right ] \\ 	 \times \frac{1[\frac{{{{{{\rm{C}}}}}}}{{{{{{\rm{s}}}}}}}]}{1000[{{{{{\rm{mA}}}}}}]}\times \frac{1[{{{{{\rm{mol}}}}}}\cdot {{{{{{\rm{e}}}}}}}^{-}]}{96485[{{{{{\rm{c}}}}}}]} \times \frac{1[{{{{{\rm{mol}}}}}}\cdot {{{{{{\rm{O}}}}}}}_{2}]}{2[{{{{{\rm{mol}}}}}}\cdot {{{{{{\rm{e}}}}}}}^{-}]}\times (6.02\times {10}^{23} \left [\frac{{{{{{\rm{atom}}}}}}\cdot {{{{{{\rm{O}}}}}}}_{2}}{{{{{{\rm{mol}}}}}}\cdot {O}_{2}} \right ])\\ 	=6.24\times {10}^{15}\left[\frac{{{{{{\rm{atom}}}}}}\cdot {{{{{{\rm{O}}}}}}}_{2}}{{{{{{\rm{s}}}}}}\cdot {{{{{\rm{c}}}}}}{{{{{{\rm{m}}}}}}}^{2}}\right]\\ 	 ({{{{{\rm{number}}}}}}\,{{{{{\rm{of}}}}}}\,{{{{{\rm{active}}}}}}\,{{{{{\rm{sites}}}}}})=0.03\left[\frac{{{{{{\rm{mg}}}}}}}{{{{{{\rm{c}}}}}}{{{{{{\rm{m}}}}}}}^{2}}\right] \\ 	 \times 0.023\times \frac{1[{{{{{\rm{mmol}}}}}}]}{58.9[{{{{{\rm{mg}}}}}}]}\times (6.02\times {10}^{20}\left[\frac{{{{{{\rm{atom}}}}}}}{{{{{{\rm{mmol}}}}}}}\right])=7.05\times {10}^{15}\left[\frac{{{{{{\rm{atom}}}}}}\cdot {{{{{\rm{Co}}}}}}}{{{{{{\rm{c}}}}}}{{{{{{\rm{m}}}}}}}^{2}}\right]$$

Therefore, the TOF (s^−1^) value for the electrochemical H_2_O_2_ production on CoPc-py-CNT is 0.89 (s^−1^). Meanwhile, the TOF values of other catalysts are calculated using the same method and compared in Supplementary Table 7.

### Flow cell performance test

0.5 M KOH was used as the aqueous electrolyte. The flow rate for the aqueous electrolyte was fixed at 2 mL min^−1^ controlled by a peristaltic pump. Solution resistance (*R*_s_) was determined by electrochemical impedance spectroscopy at frequencies ranging from 0.1 Hz to 100 kHz. All the measured potentials using a three-electrode setup were manually 100% *iR*-compensated. The cathode electrode was prepared by uniformly coating the well-mixed catalyst ink on the natural air diffusion electrode, which was prepared using the carbon felt coated with PTFE and calcined at 350 °C for 1 h. The catalyst ink was prepared with catalyst powder, PTFE suspension, isopropanol, binder (0.25 wt% Nafion-ethanol) with the mass ratios to be 1: 0.1: 20: 2, followed by sonication for 30 min until the solution is uniformly dispersed. The loading of catalyst at the cathode is 0.5 mg cm^−2^. Nickel mesh was used as the anode electrode to construct the flow cell system. The proton exchange membrane (Nafion 117) was used to separate the cathode and anode. For the stability test, the chronopotentiometry method was used with the current fixed at 300 mA (electrode surface area = 1 cm^2^). The electrolyte was sampled every 2 h to determine the Faradaic efficiency and the H_2_O_2_ concentration, and the electrolyte was refreshed at the same time interval. 5 mM EDTA was added into the catholyte to stabilize the produced H_2_O_2_. The cell voltage of flow cell at 300 mA cm^−2^ current density is ~6.5 V during the stability test.

### Computational methods

Spin-polarized DFT calculations were performed using the Vienna Ab initio Simulation Package^48,49^. The generalized gradient approximation with the PBE functional was used to describe the exchange and correlation energy^50^. Electron−ion interactions were treated by the projector augmented wave method^51^. The DFT + U method was applied to the 3d orbitals of transition metals to correct for artificial electron self-interaction^52^. The U values for the 3d transition metals are taken from reference^53^, which were developed to reproduce the experimental heat of the formation of transition-metal oxides. Brillouin zone was sampled by Monkhorst-Pack k-point meshes^54^. Optimized structures were obtained by minimizing the forces on each ion until they fell below 0.05 eV/Å.

The energetics of the electrochemical ORR was calculated with the computational hydrogen electrode method^55^. Briefly, the Gibbs free energy change of each electrochemical elementary step of the ORR was calculated with DFT. The 4e^−^ ORR reaction mechanism was assumed to follow the four-step associative mechanism represented by:

14 $${{{{{{\rm{O}}}}}}}_{2}+{{{{{{\rm{H}}}}}}}^{+}+{{{{{{\rm{e}}}}}}}^{-}+\ast \leftrightarrow {{{{{{\rm{OOH}}}}}}}^{*}$$

15 $${{{{{{\rm{OOH}}}}}}}^{*}+{{{{{{\rm{H}}}}}}}^{+}+{{{{{{\rm{e}}}}}}}^{-}\leftrightarrow {{{{{{\rm{O}}}}}}}^{*}+{{{{{{\rm{H}}}}}}}_{2}{{{{{\rm{O}}}}}}$$

16 $${{{{{{\rm{O}}}}}}}^{*}+{{{{{{\rm{H}}}}}}}^{+}+{{{{{{\rm{e}}}}}}}^{-}\leftrightarrow {{{{{{\rm{OH}}}}}}}^{*}$$

17 $${{{{{{\rm{OH}}}}}}}^{*}+{{{{{{\rm{H}}}}}}}^{+}+{{{{{{\rm{e}}}}}}}^{-}\leftrightarrow {{{{{{\rm{H}}}}}}}_{2}{{{{{\rm{O}}}}}}+\ast$$

The 2e^−^ reaction pathway producing H_2_O_2_ is:

18 $${{{{{{\rm{O}}}}}}}_{2}+{{{{{{\rm{H}}}}}}}^{+}+{{{{{{\rm{e}}}}}}}^{-}+\ast \leftrightarrow {{{{{{\rm{OOH}}}}}}}^{*}$$

19 $${{{{{{\rm{OOH}}}}}}}^{*}+{{{{{{\rm{H}}}}}}}^{+}+{{{{{{\rm{e}}}}}}}^{-}\leftrightarrow {{{{{{\rm{H}}}}}}}_{2}{{{{{{\rm{O}}}}}}}_{2}$$

The free energy change of each elementary step is calculated as $\triangle {{\mbox{G}}}\,=\,\triangle {{\mbox{E}}}-{{\mbox{T}}}\triangle {{\mbox{S}}}+\triangle {{\mbox{ZPE}}}$, where $\triangle {{\mbox{E}}}$ is the total energy change, $\triangle {{\mbox{ZPE}}}$ is the change in zero-point energy, and ${{\mbox{T}}}\triangle {{\mbox{S}}}$ is entropic change. All free energies are calculated relative to H_2_O (l) and H_2_ (g). The changes in $\triangle {{\mbox{ZPE}}}$ and ${{\mbox{T}}}\triangle {{\mbox{S}}}$ are calculated using previously determined values. The chemical potential of the solvated proton and electron pair (H^+^ + e^−^) at standard conditions (pH = 0, *T* = 298.15 K) is calculated as 1/2 G_H2_ + eU_SHE_ assuming equilibrium at the standard hydrogen electrode. The solvation effects were also considered using an implicit solvation model implemented in VASPsol^56,57^. The relative permittivity for the continuum solvent was set to 80 to simulate a water environment. For the 4e^−^ and 2e^−^ pathways, the equilibrium potentials at standard conditions are 1.23 and 0.68 V_SHE_, respectively.

## Supplementary information

Supplementary Information

## Data Availability

Data sets generated during and/or analyzed during the current study are available from the first authors and corresponding authors on request. Source data are provided with this paper.

## Source data

Source data
