# Supplementary material for: Rational design of heterogenized molecular phthalocyanine hybrid single-atom electrocatalyst towards two-electron oxygen reduction
Source: Nat Commun. 2023 Mar 14;14:1426. doi: 10.1038/s41467-023-37066-y (PMC10014850; doi:10.1038/s41467-023-37066-y)
Supplement: Supplementary file 1 — Supplementary Information [file 41467_2023_37066_MOESM1_ESM.pdf]

Supplementary Information

Rational design of heterogenized molecular phthalocyanine hybrid single-atom electrocatalyst towards two-electron oxygen reduction

Wenjun Fan,[1] Zhiyao Duan,*[2] Wei Liu,[3] Rashid Mehmood,[1,4] Jiating Qu,[1,4] Yucheng Cao,[1,4] Xiangyang Guo,[1] Jun Zhong,[5] and Fuxiang Zhang*[1]

[1] *State Key Laboratory of Catalysis, Dalian National Laboratory for Clean Energy, Dalian Institute of Chemical Physics, Chinese Academy of Sciences, Dalian, 116023, China.*

[2] *State Key Laboratory of Solidification Processing, School of Materials Science and Engineering, Northwestern Polytechnical University, Xi'an 710072, P. R.China.*

[3] *State Key Laboratory of Fine Chemicals, School of Chemical Engineering, Dalian University of Technology, Dalian, 116024 China.*

[4] *University of Chinese Academy of Sciences; Beijing, 100049, China.*

[5] *Institute of Functional Nano and Soft Materials (FUNSOM), Jiangsu Key Laboratory for Carbon-Based Functional Materials and Devices, Soochow University, Suzhou 215123, Jiangsu (P. R. China)*

***Corresponding author:** *zhiyao.duan@nwpu.edu.cn (Z.D.), fxzhang@dicp.ac.cn (F.Z.)*

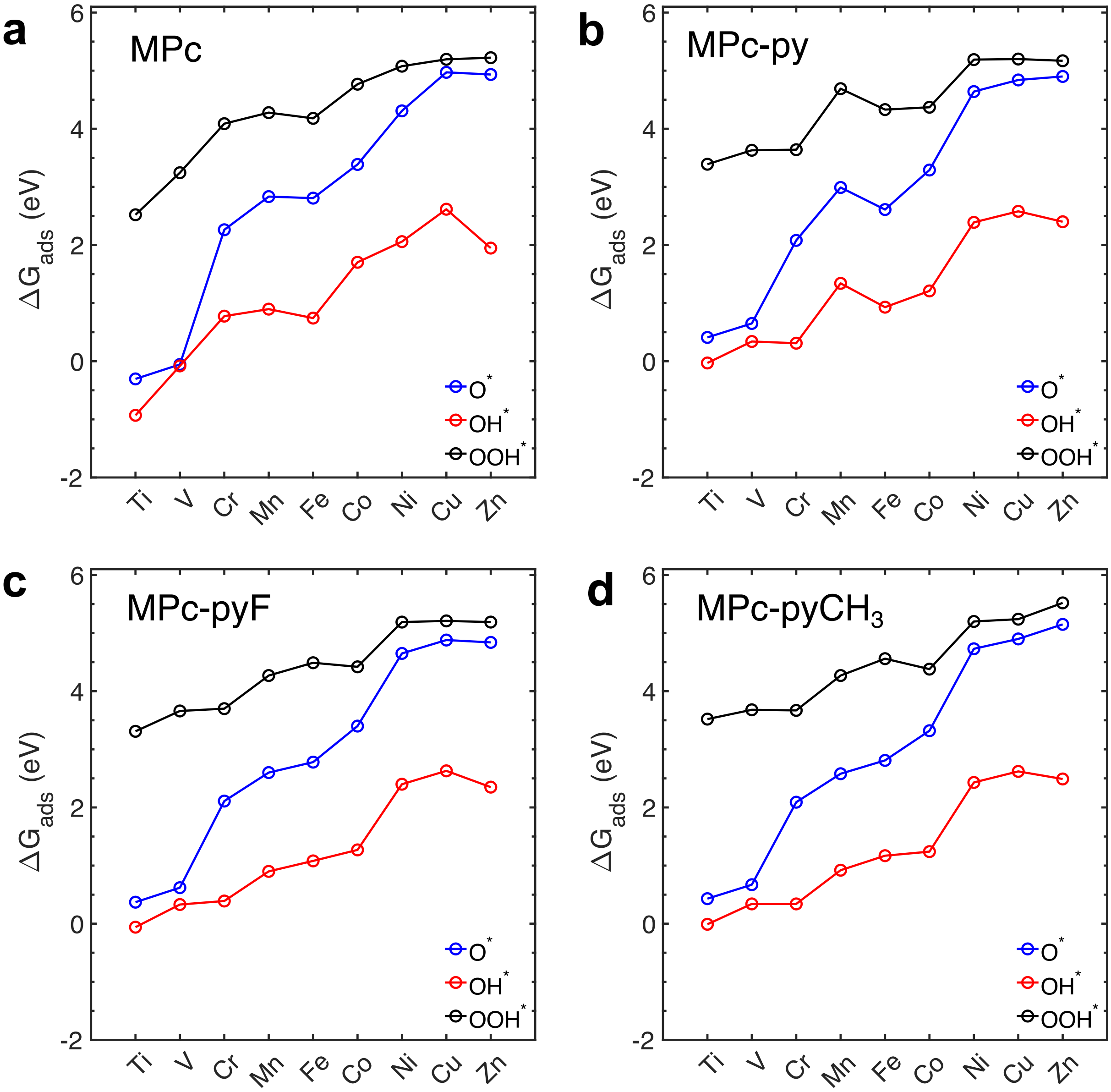

Supplementary Fig. 1. Calculated free energies of adsorption of ORR intermediates on **a,** MPc, **b,** M HSACs, **c,** M HSACs-F, **d,** M HSACs-CH_3 (M = Ti, V, Cr, Mn, Fe, Co, Ni, Cu, Zn). Source data are provided as a Source Data file.

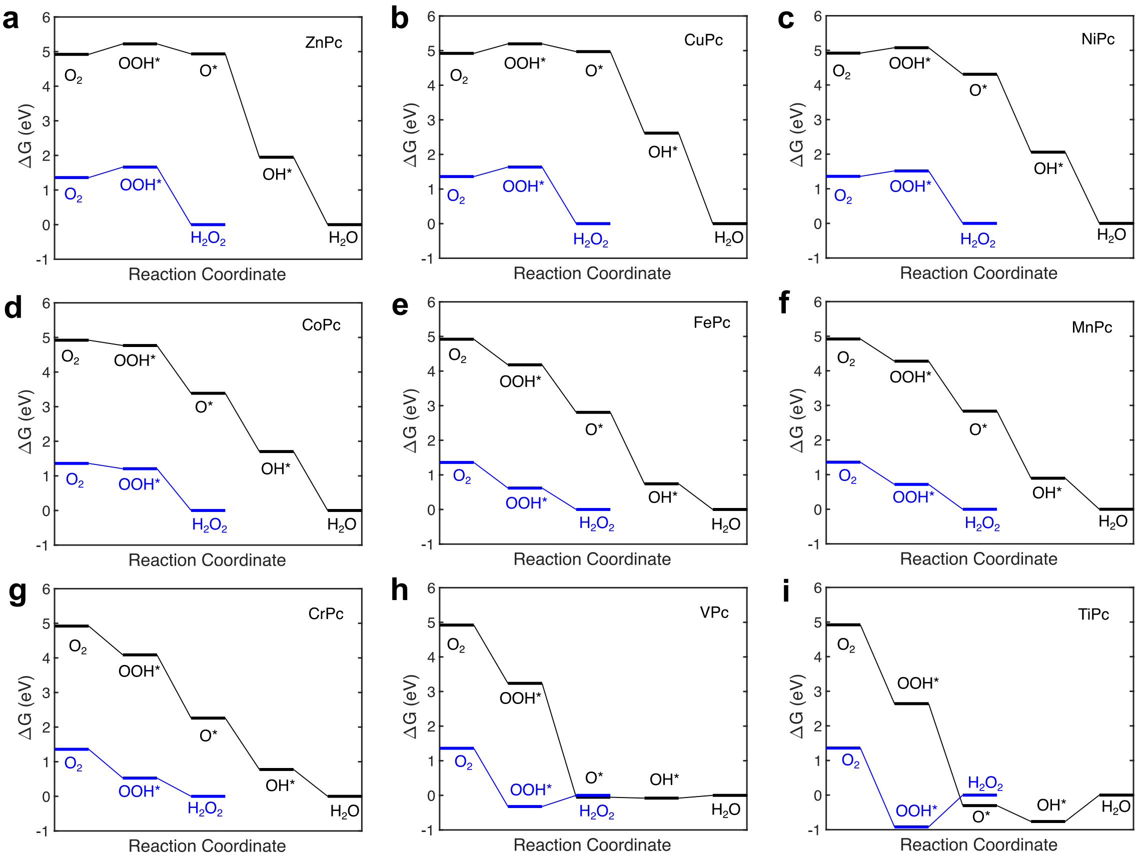

Supplementary Fig. 2. Calculated reaction free energy diagrams of ORR to H_2O_2 (blue) and water (black) on MPc at U = 0 V_{RHE} (M = Ti, V, Cr, Mn, Fe, Co, Ni, Cu, Zn). Source data are provided as a Source Data file.

Apparently, MnPc favors the O-OH bond splitting for full ORR over the H_2O_2 production due to the lower free change of the former. On the contrary, CoPc has a ΔG_{OOH*} slightly weaker than 4.6 eV, thus the ORR reaction should proceed via the 2e$^-$ reduction pathway. Interestingly, the optimal activity for the H_2O_2 production is predicted to occur at ΔG_{OOH*}~4.2 eV by the volcano plot (see Figure 1c). But if we further consider the energetics of the competing step for the optimal selectivity as discussed above, the ΔG_{OOH*} needs to be further destabilized to about 4.6 eV in order to make H_2O_2 production thermodynamically favorable than the competing O* generation step. In this regard, CoPc should have the highest selectivity for the H_2O_2 production while still maintain appreciable ORR activity among all MPc catalysts studied.

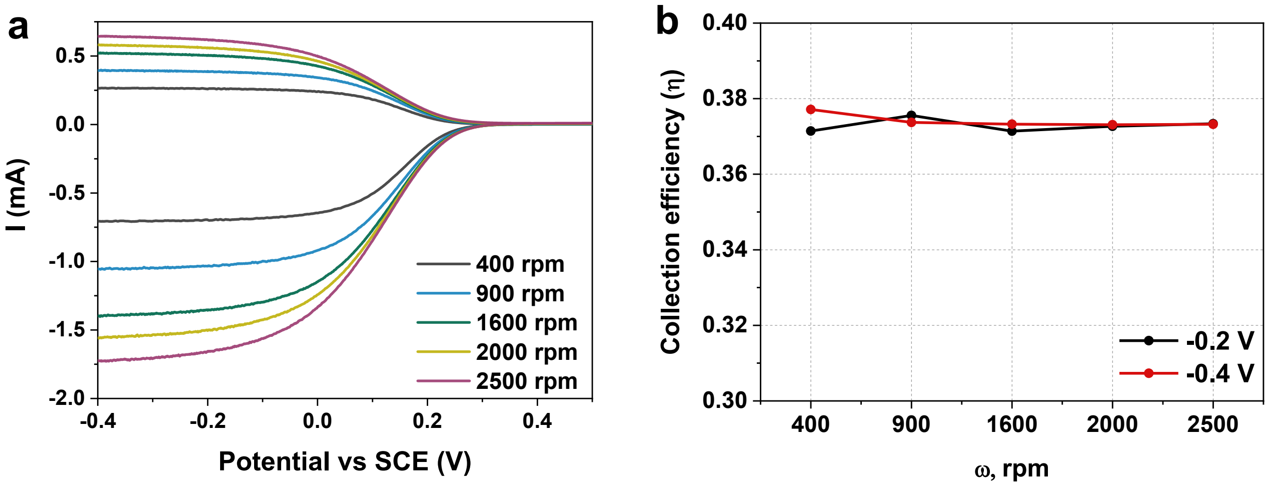

Supplementary Fig. 3. Determination of correction efficiency of RRDE with $K_3Fe[CN]_6$. LSVs of a Ar-saturated aqueous solution of $K_3Fe[CN]_6$ (10 mmol L^{-1}) in the presence of KNO_3 (0.1 M) recorded at a rotating-ring (Pt) and disk (GC) electrode. (a) RRDE voltammograms. The disk potential was scanned at 10 mV s^{-1} and the ring potential was constant at 0.9 V vs SCE. (b) Plots of collection efficiency at -0.2 V and -0.4V vs rotation rates. Source data are provided as a Source Data file.

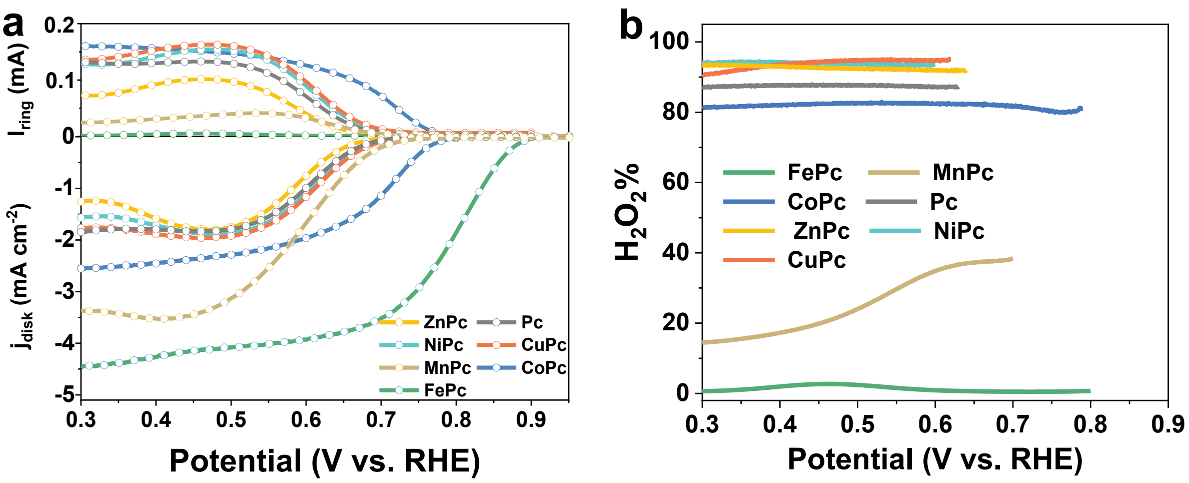

Supplementary Fig. 4. Electrochemical 2e$^-$ ORR performance of various MPc. a, RRDE voltammograms of MPc in O_2-saturated solution in 0.1 M KOH, showing the disk current density (j_{disk}) and ring current (i_{ring}). **b,** Calculated H_2O_2 selectivity (H_2O_2%) as a function of applied potential. Source data are provided as a Source Data file.

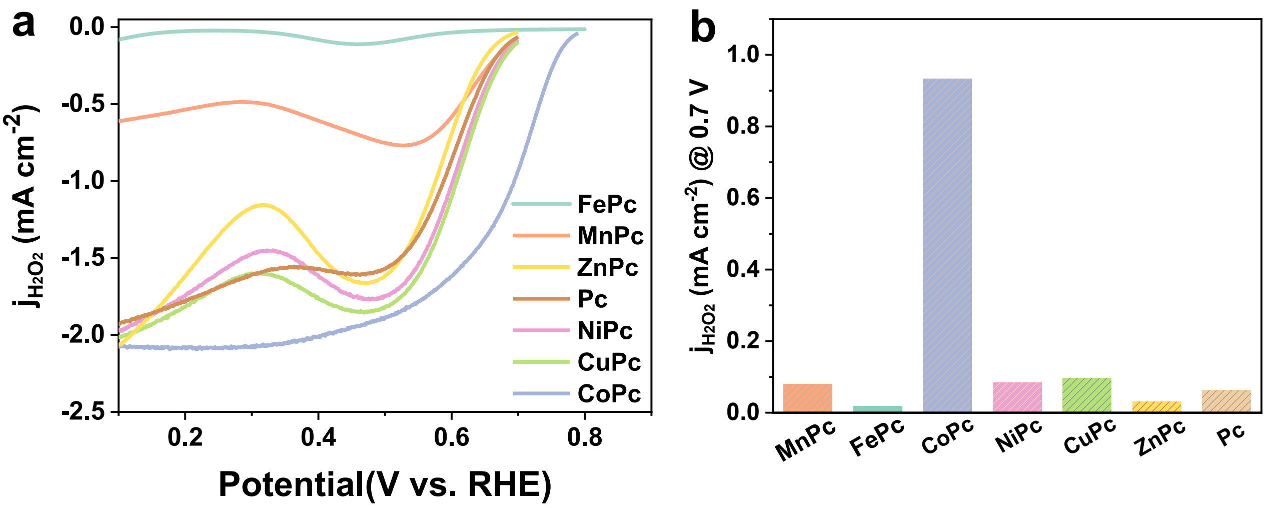

Supplementary Fig. 5. a, Partial current density for H_2O_2 production on various MPc obtained from the ring current in Supplementary Fig. 4a. **b,** Comparison of H_2O_2 partial current densities at 0.7 V for MPc. Source data are provided as a Source Data file.

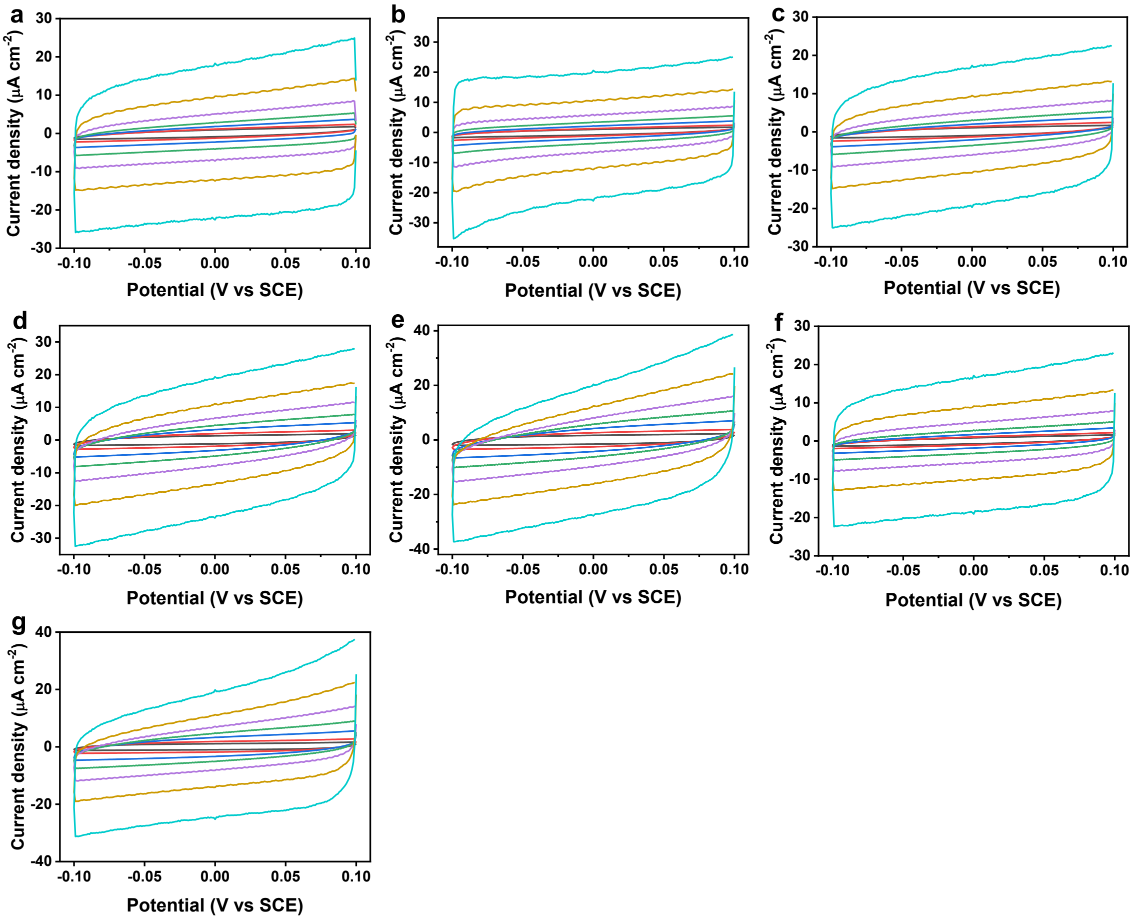

Supplementary Fig. 6. Double-layer capacitance measurements for determining electrochemically active surface area for the MPc and Pc from voltammetry in 0.1 M KOH. Cyclic voltammograms were measured in a non-Faradaic region of the voltammogram at the following scan rate: decrease in the order 0.8, 0.4, 0.2, 0.1, 0.05, 0.02, and 0.01 V/s. The working electrode was held at each potential vertex for 10 s before the beginning of the next sweep. All current is assumed to be due to capacitive charging. **a,** MnPc, **b,** FePc, **c,** CoPc, **d,** NiPc, **e,** CuPc, **f,** ZnPc, **g,** Pc. Source data are provided as a Source Data file.

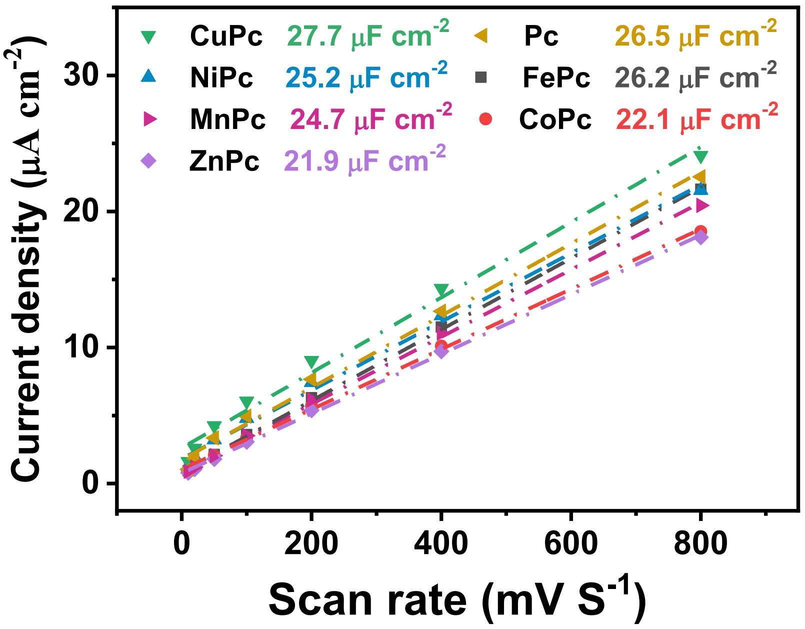

Supplementary Fig. 7. Double-layer capacitance for the MPc and Pc from cyclic voltammetry in Supplementary Fig. 6. The cathodic and anodic charging currents measured at 0.0 V vs SCE were plotted as a function of scan rate. Source data are provided as a Source Data file.

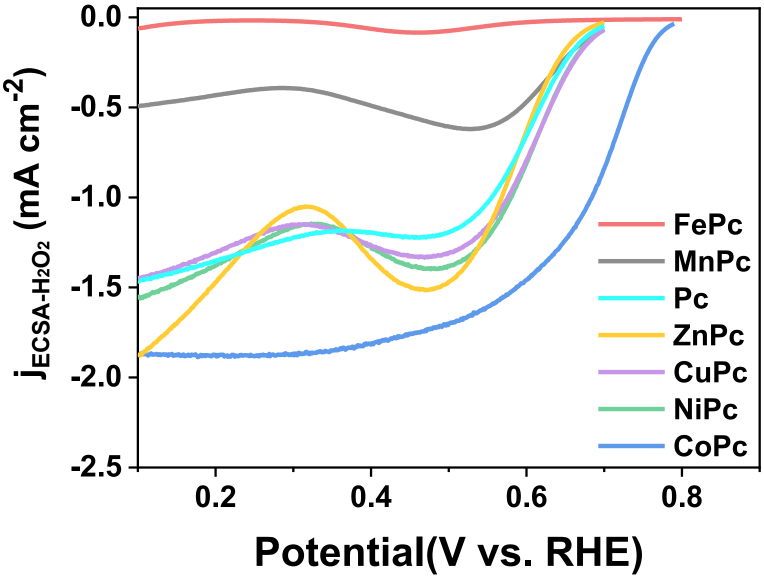

Supplementary Fig. 8. The ECSA-normalized current density for H_2O_2 production on MPc in O_2-saturated 0.1 M KOH solution, rotation speed 1600 rpm. Source data are provided as a Source Data file.

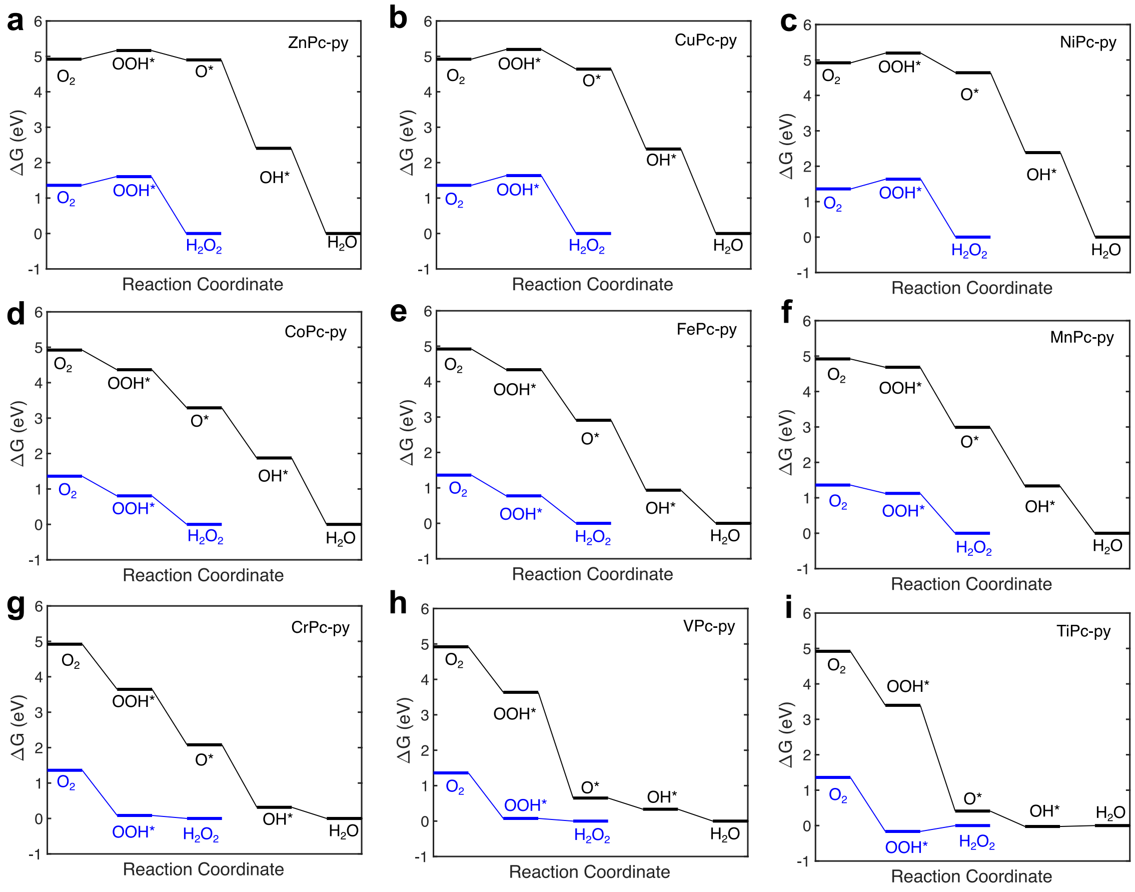

Supplementary Fig. 9. Calculated reaction free energy diagrams of ORR to H_2O_2 (blue) and water (black) on M HSACs at U = 0 V_{RHE} (M = Ti, V, Cr, Mn, Fe, Co, Ni, Cu, Zn). Source data are provided as a Source Data file.

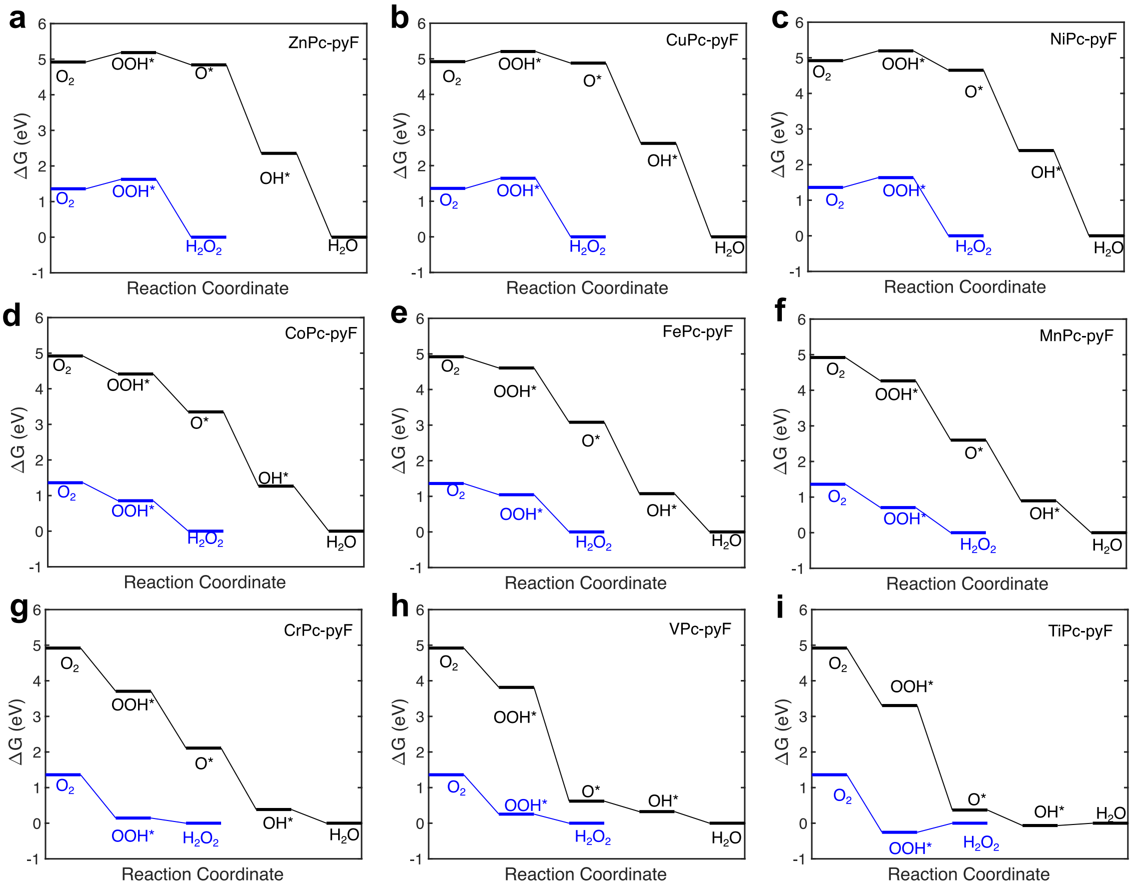

Supplementary Fig. 10. Calculated reaction free energy diagrams of ORR to H_2O_2 (blue) and water (black) on M HSACs-F at U = 0 V_{RHE} (M = Ti, V, Cr, Mn, Fe, Co, Ni, Cu, Zn). Source data are provided as a Source Data file.

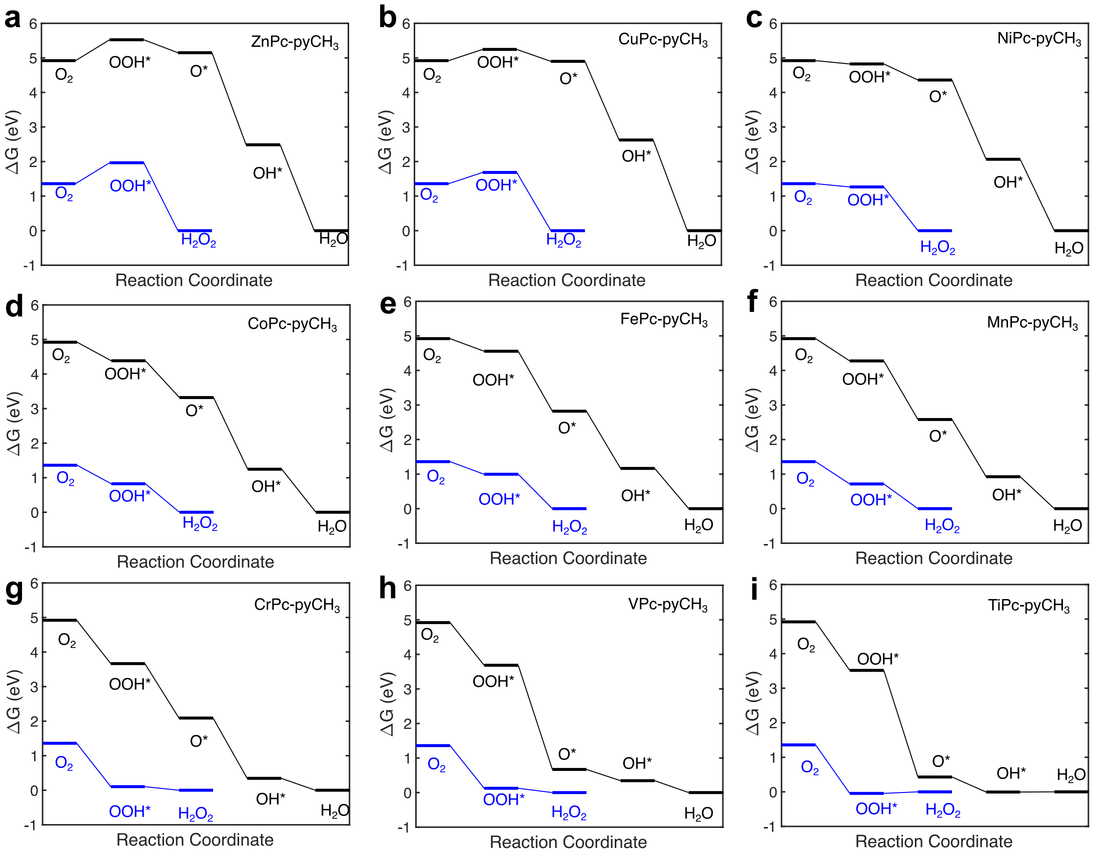

Supplementary Fig. 11. Calculated reaction free energy diagrams of ORR to H_2O_2 (blue) and water (black) on M HSACs-CH_3 at U = 0 V_{RHE} (M = Ti, V, Cr, Mn, Fe, Co, Ni, Cu, Zn). Source data are provided as a Source Data file.

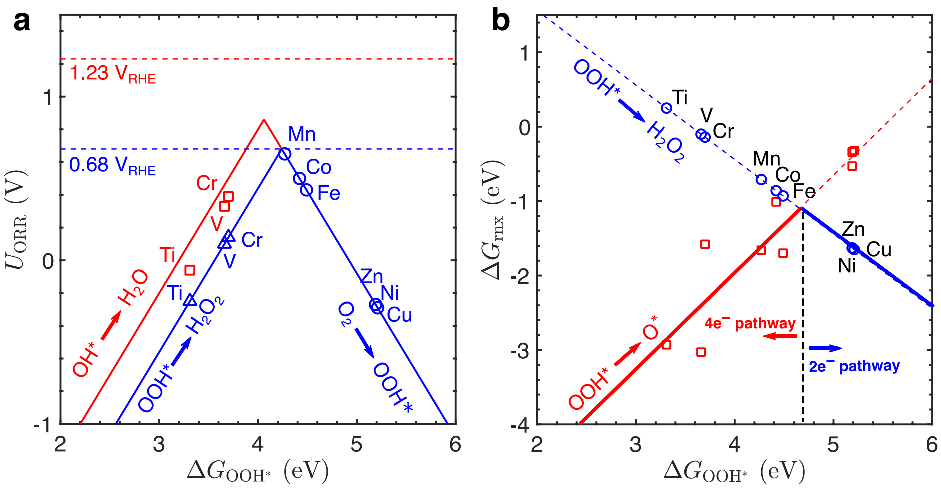

Supplementary Fig. 12. a, Calculated catalytic activity volcano plot for the $2e^-$ and $4e^-$ reaction pathways of ORR over M HSACs-F. **b,** Calculated selectivity plot of ORR over M HSACs-F (M = Ti, V, Cr, Mn, Fe, Co, Ni, Cu, Zn). Source data are provided as a Source Data file.

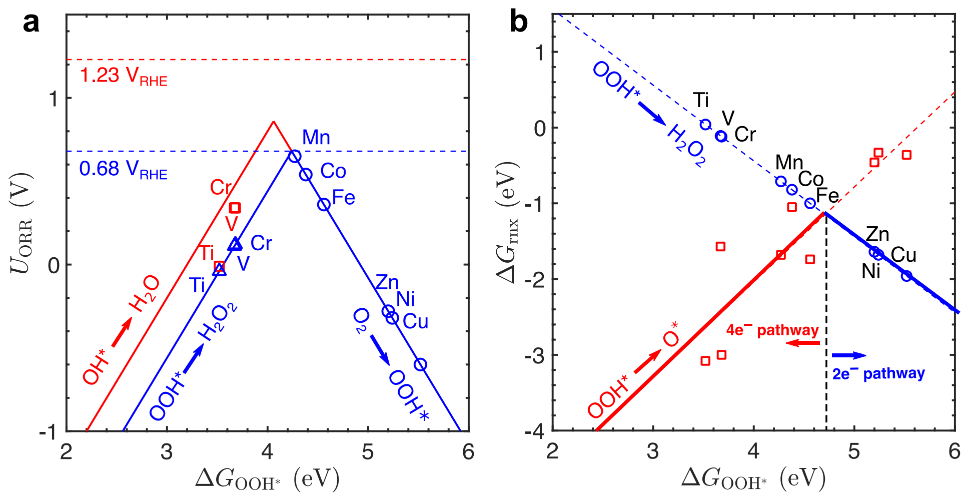

Supplementary Fig. 13. a, Calculated catalytic activity volcano plot for the $2e^-$ and $4e^-$ reaction pathways of ORR over M HSACs-CH_3. **b,** Calculated selectivity plot of ORR over M HSACs-CH_3 (M = Ti, V, Cr, Mn, Fe, Co, Ni, Cu, Zn). Source data are provided as a Source Data file.

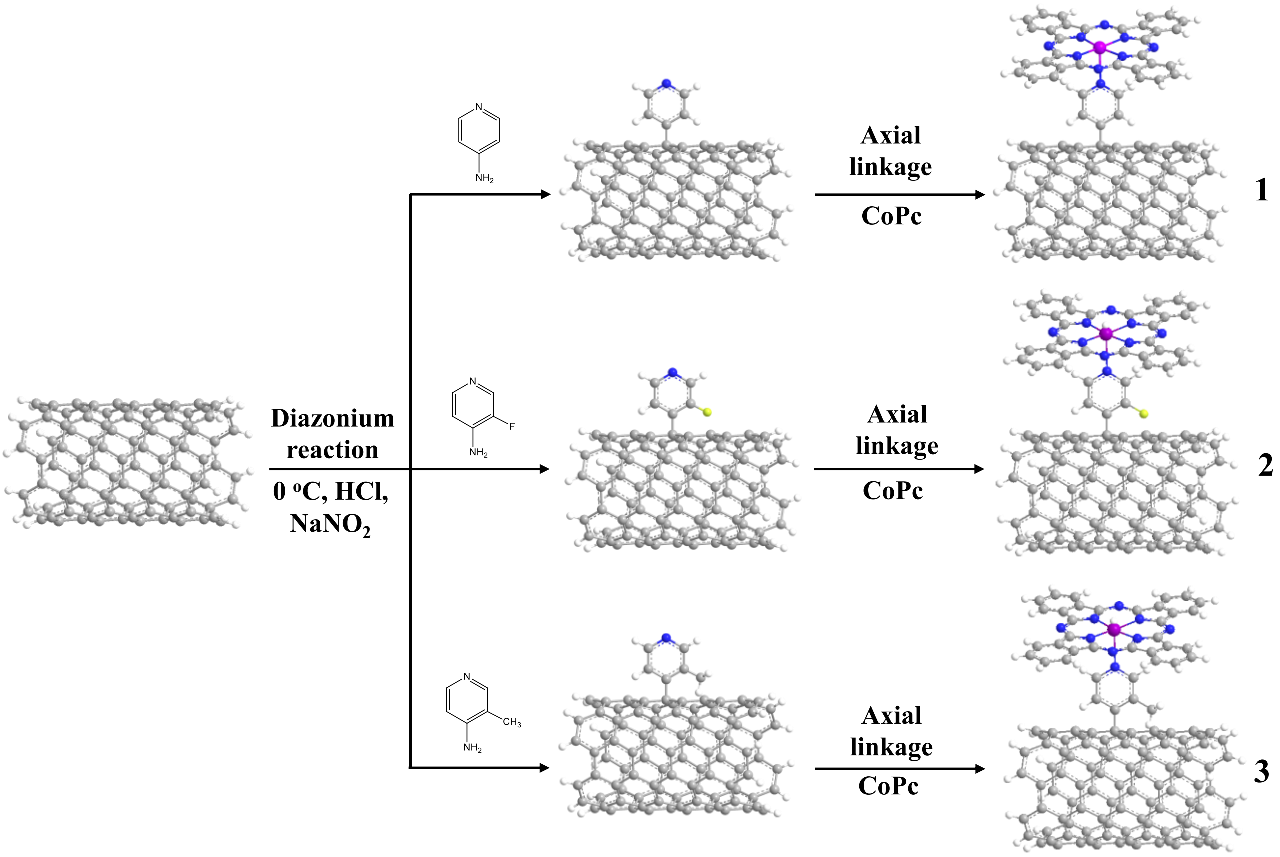

Supplementary Fig. 14. The schematic diagram for the synthesis of heterogenized CoPc catalyst with CNT as support. 1: Co HSACs; 2: Co HSACs-F; 3: Co HSACs-CH_3. Firstly, CNT was functionalized with aminopyridine by the diazonium reaction. Afterward, CoPc is axially coordinated on CNT through the metal-ligand bond between Co and N.

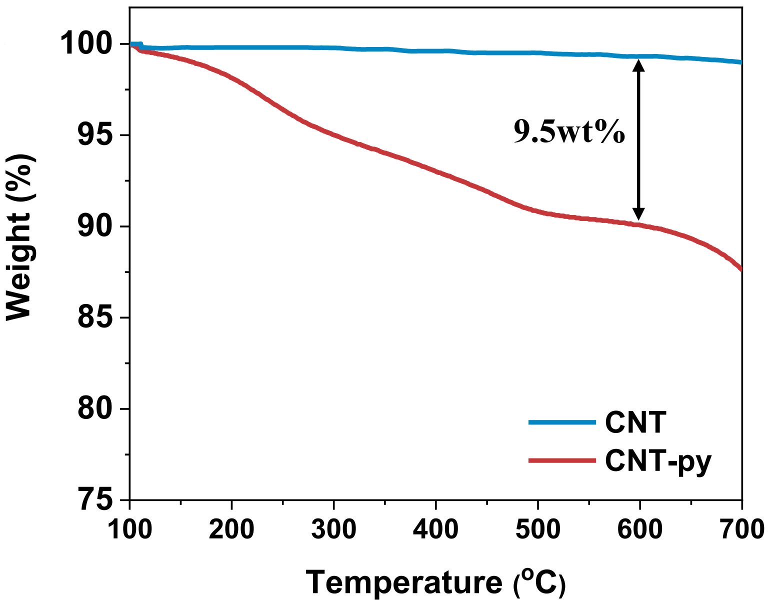

Supplementary Fig. 15. TGA data (10 °C min^{-1}) of purified CNT and pyridine-functionalized CNT (CNT-py). Source data are provided as a Source Data file.

Note:

The covalent functionalization of CNT by pyridine group was examined by TGA analysis, which showed a weight loss of 9.5 wt% at 600 °C compared to nearly no change for the purified CNT. This corresponds to ca.1 functional group for every 60 nanotube carbon atoms.

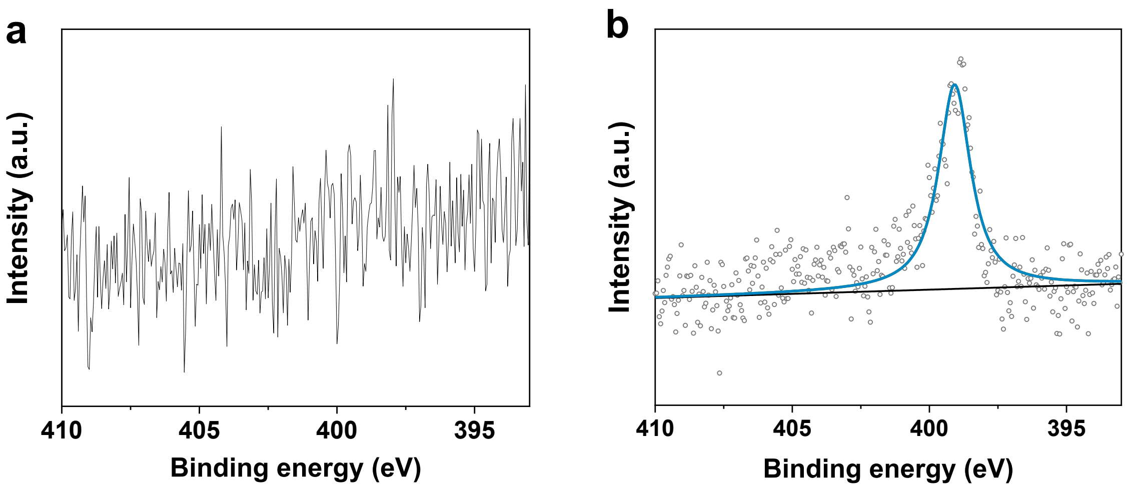

Supplementary Fig. 16. N1*s* XPS spectra. a, CNT. **b,** pyridine-functionalized CNT (CNT-py). The bare CNT shows no N1*s* signal, while CNT-pyridine exhibits a significant N1*s* peak ascribing to pyridine N at ca.399 eV. Source data are provided as a Source Data file.

Note:

The functionalization of CNT with pyridine group was further confirmed by XPS measurements, where elemental composition analysis exhibited the presence of ca. 1.5 at% of N in CNT-py (Supplementary Table 6), in agreement with the TGA data.

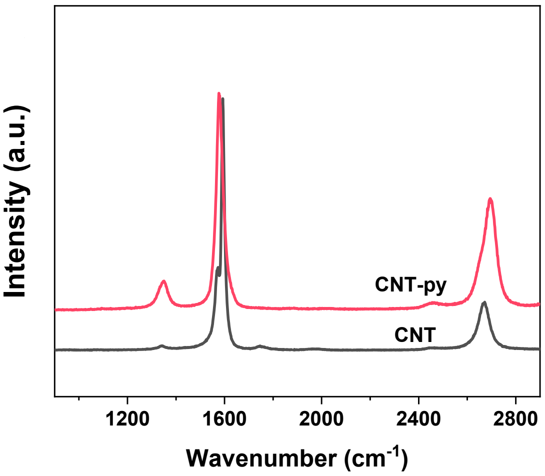

Supplementary Fig. 17. Raman spectra (532 nm) of purified CNT and pyridine-functionalized CNT (CNT-py) normalized at the G-band. Source data are provided as a Source Data file.

Note:

Generally, the D band accounts for the presence of defects and the G band represent the graphitic order. The Raman spectra of CNT-py exhibits an enhanced D band at ca. 1330 cm^{-1} compared with that of the pure CNT, indicative of groups attached to the surface of the nanotubes.

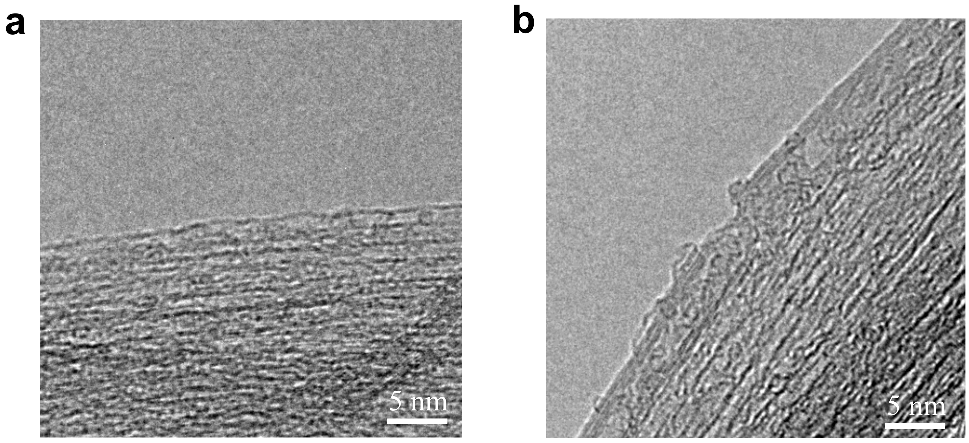

Supplementary Fig. 18. HRTEM characterization. a, CNT. **b,** Co HSACs. Source data are provided as a Source Data file.

Note:

The HRTEM image in Supplementary Fig.18b shows surface bumps along the sidewalls of CNT, which is the evidence of covalently functionalized moieties of pyridine group attached to CNT.

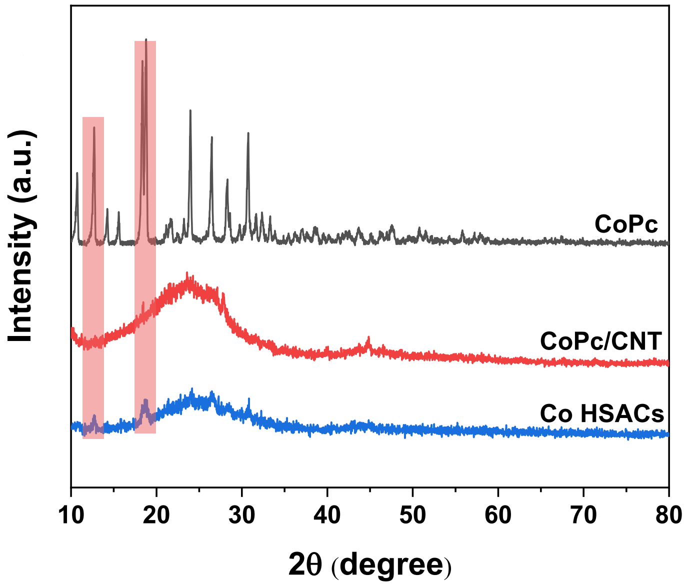

Supplementary Fig. 19. XRD patterns of the CoPc, CoPc/CNT, and the Co HSACs. Source data are provided as a Source Data file.

Note: XRD patterns reveal both Co HSACs and CoPc/CNT are comprised of carbon and CoPc.

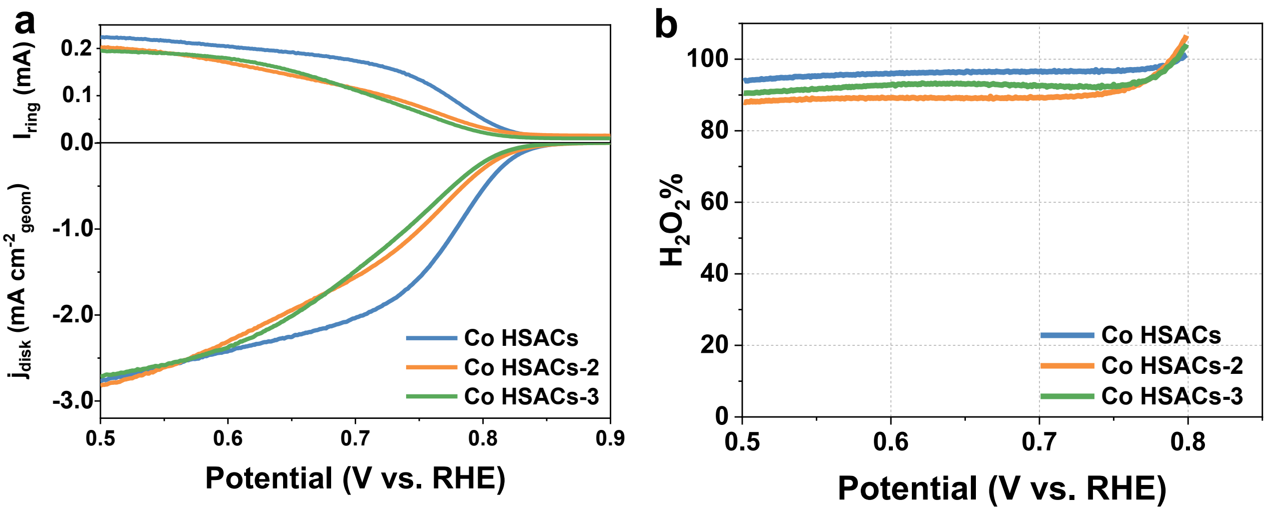

Supplementary Fig. 20. Optimization of the content of CoPc in the Co HSACs. a, Comparison of 2e$^-$ ORR performance at 1600 rpm and the simultaneous H_2O_2 detection current densities at the ring electrode for Co HSACs, Co HSACs-2, and Co HSACs-3 in 0.1 KOH. **c,** The calculated H_2O_2 selectivity as a function of the applied potential. The contents of Co in Co HSACs, Co HSACs-2, and Co HSACs-3 are 0.7at%, 1.4 at%, and 2.1at%, respectively. Source data are provided as a Source Data file.

Note:

Considering that the N content which corresponds to the pyridinic group on the CNT is 1.43 at% by XPS (Supplementary Table 6), we have examined the effect of pyridine group content in the CNT-py using three different values, i.e., 0.7 at%, 1.4 at%, and 2.1 at% to determine the optimal loading of CoPc in the HSACs. For simplicity, the three samples with different additive amount of CoPc were denoted as Co HSACs, Co HSACs-1, and Co HSACs-2, respectively. As shown in Supplementary Fig. 20, the Co HSACs delivers higher 2e$^-$ activity with more positive onset potential (Supplementary Fig. 20a) as well as better 2e$^-$ selectivity (Supplementary Fig. 20b) than the other two samples. Although the Co HSACs with CoPc loading of 1.4 at% and 2.1 at% are higher than the 0.7 at% one, the excess CoPc may not coordinate with the CNT-py via the pyridinic group, due to the number of effective coordination sites and steric effect. As a result, only the coordinated CoPc could prominently enhance the 2e$^-$ performance. Owing to the superior 2e$^-$ performance of Co HSACs, we thus chose the 0.7 at% content of CoPc for the discussion in our manuscript.

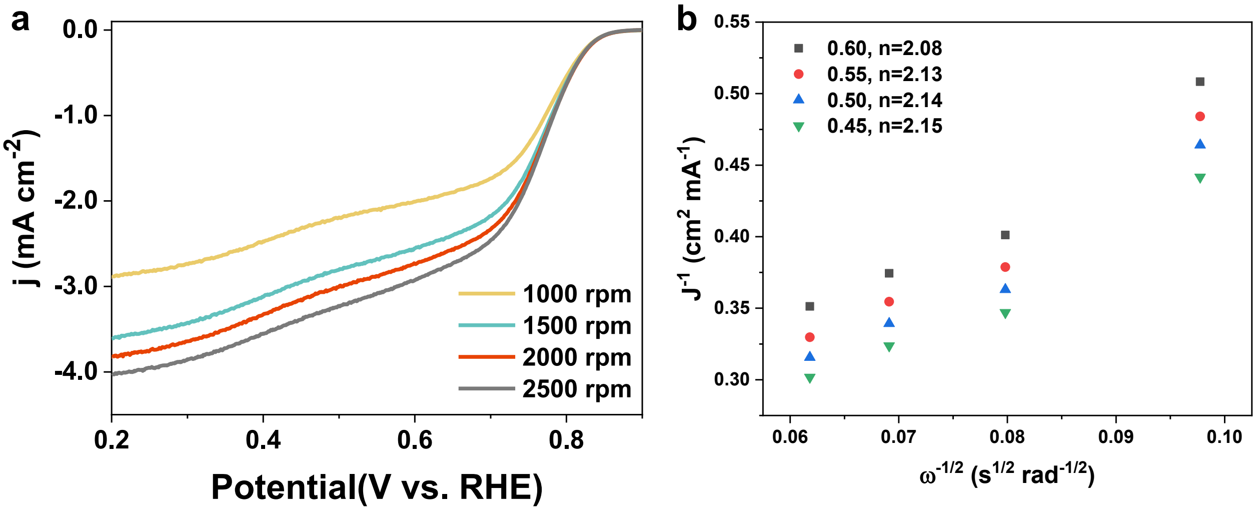

Supplementary Fig. 21. a, J-V curve of Co HSACs electrode measured in O_2-saturated with different rotating rates. **b,** K-L plots at different potentials calculated from the LSVs with different rotating rates. Source data are provided as a Source Data file.

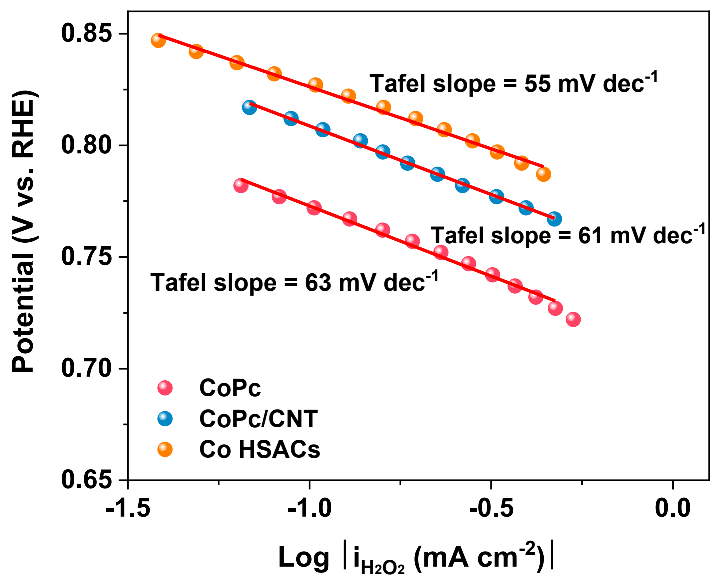

Supplementary Fig. 22. Tafel plots of the typical samples. Source data are provided as a Source Data file.

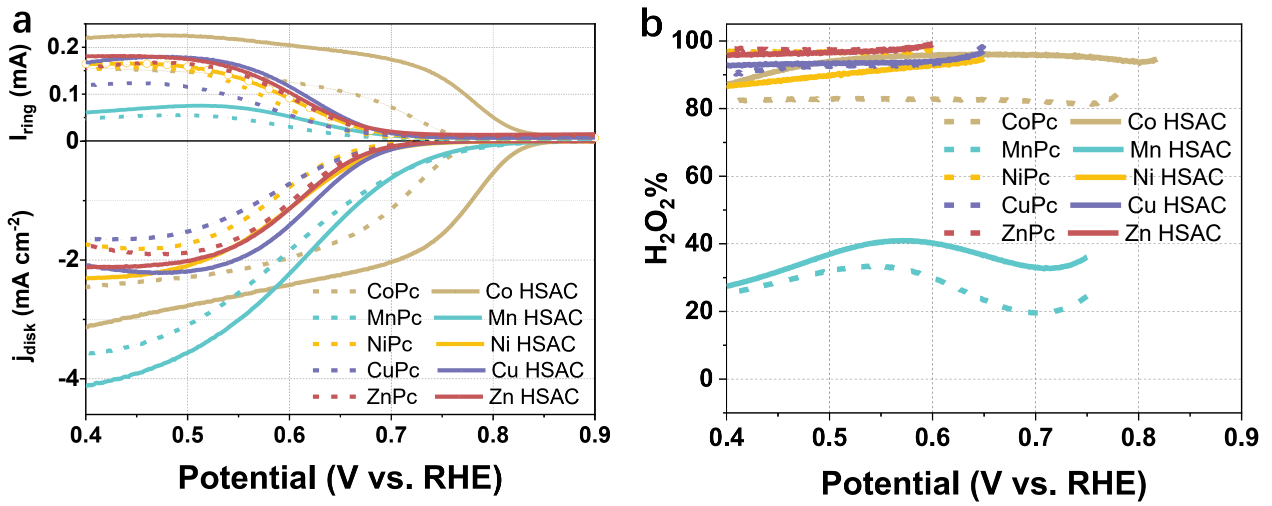

Supplementary Fig. 23. Electrochemical 2e$^-$ ORR performance of various MPc and M HSACs. **a,** RRDE voltammograms of MPc and M HSACs in O_2-saturated solution in 0.1 M KOH, showing the disk current density (j_{disk}) and ring current (i_{ring}). **b,** Calculated H_2O_2 selectivity (H_2O_2%) as a function of applied potential. Source data are provided as a Source Data file.

Note:

We prepared a series of M HSACs (M = Mn, Ni, Fe, and Zn) besides Co HSACs using the similar synthesis protocol to that of the Co HSACs. As shown in Supplementary Fig. 23, all the M HSACs show higher ORR activities as compared with the MPc. Furthermore, the 2e$^-$ selectivities of M HSACs are maintained as compared with those of the MPc counterparts (Figure Supplementary Fig. 23b), indicating the general applicability by heterogenization of the MPc to generate the HSACs for enhanced ORR performance and the capability of generating H_2O_2 is anticipated to increase on the M HSACs.

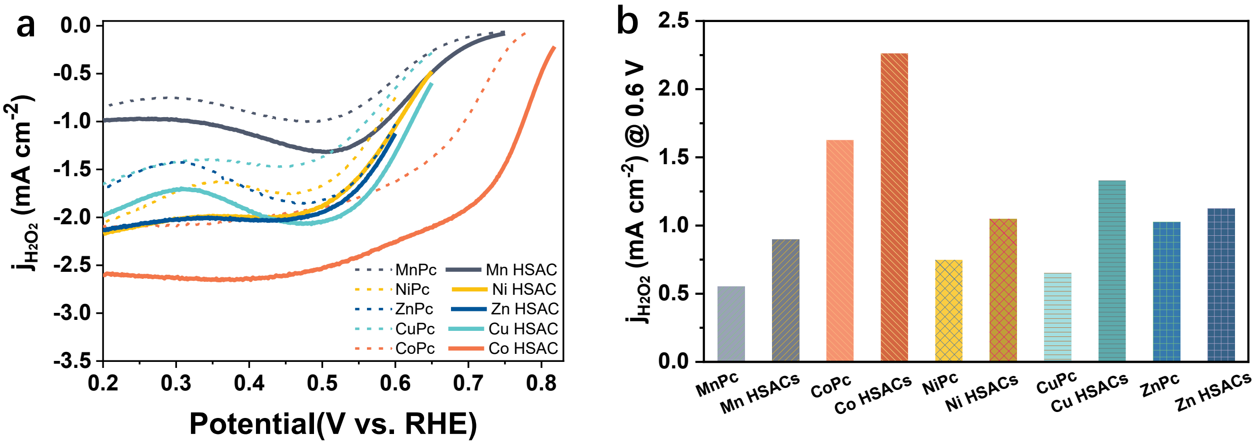

Supplementary Fig. 24. a, Partial current density for H_2O_2 production on various MPc and M HSACs obtained from the ring current in Figure R5. **b,** Comparison of H_2O_2 partial current densities at 0.6 V for MPc and M HSACs. Source data are provided as a Source Data file.

Note:

To prove whether the Mn-, Zn-, Ni-, and Cu-based HSACs are better catalysts for H_2O_2 production with high selectivity and compensated activity, we further calculated the partial current densities for H_2O_2 (j_{H2O2}) on various M HSACs for H_2O_2 production from the ring current. As shown in Supplementary Fig. 24, the Co HSACs exhibits a much earlier onset potential for H_2O_2 generation and its j_{H2O2} is prominently higher than other samples across the whole range of potential windows. Specially, the j_{H2O2} on Co HSACs at 0.6 V is ca. 2.3 mA cm^{-2}, which is 1.4 times, 2.6 times, 2.5 times, 1.7 times, and 2.0 times as those of CoPc, Mn HSACs, Ni HSACs, Cu HSACs, and Zn HSACs, respectively. Therefore, it is reasonable to conclude that the Co HSACs could deliver the best H_2O_2 production ability, although the Cu-, Ni-, and Zn-based HSACs also exhibit high selectivity for H_2O_2 production.

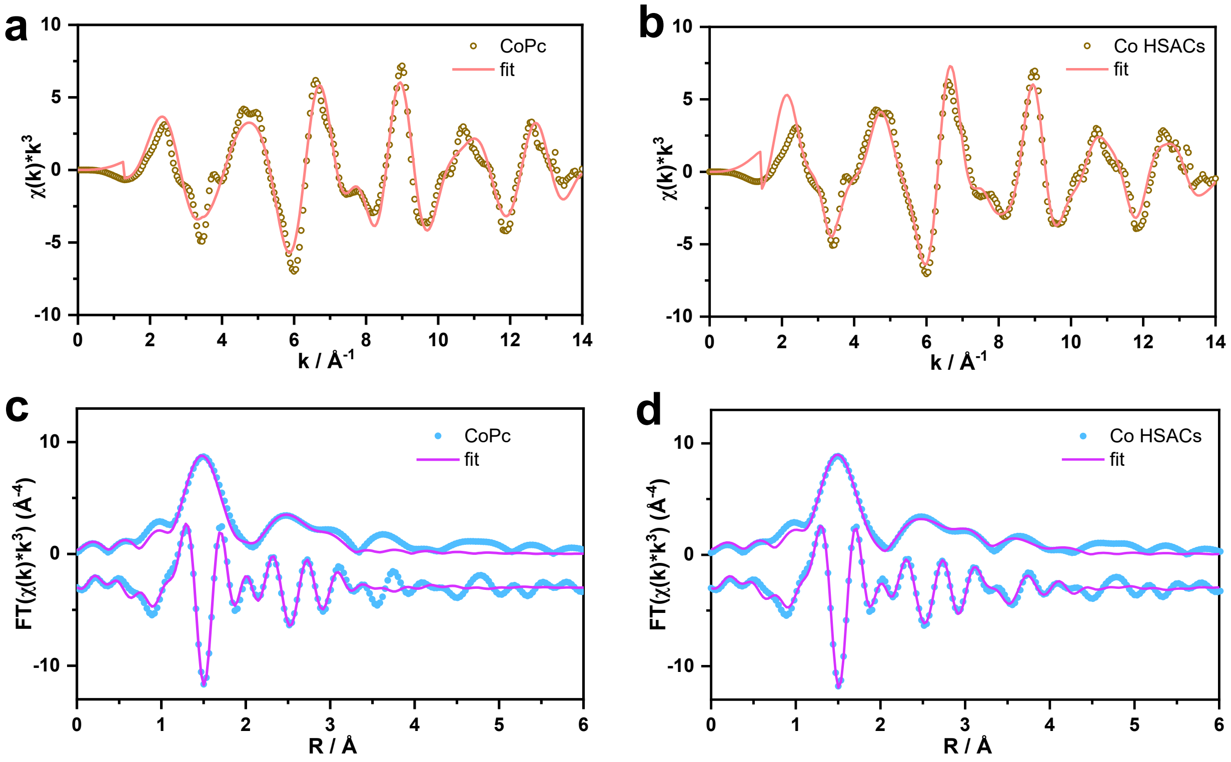

Supplementary Fig. 25. Fitting curves of the FT-EXAFS spectra for CoPc and Co HSACs: a-b, at K space. **c-d,** at R space. Source data are provided as a Source Data file.

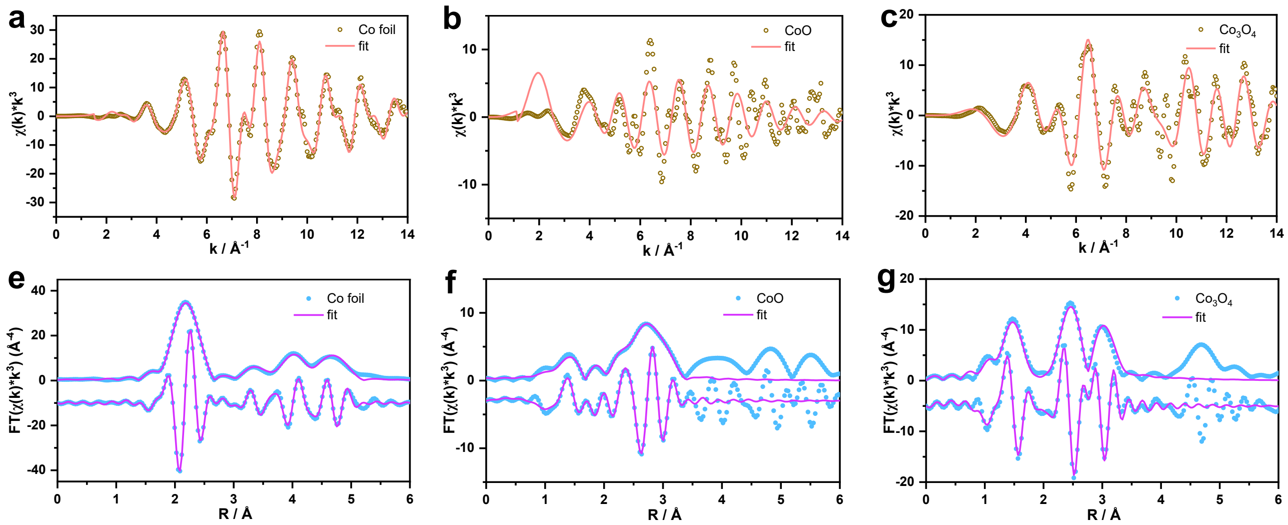

Supplementary Fig. 26. Fitting curves of the FT-EXAFS spectra for Co foil, CoO, and Co_3O_4: a-c, at K space. **c-d,** at R space. Source data are provided as a Source Data file.

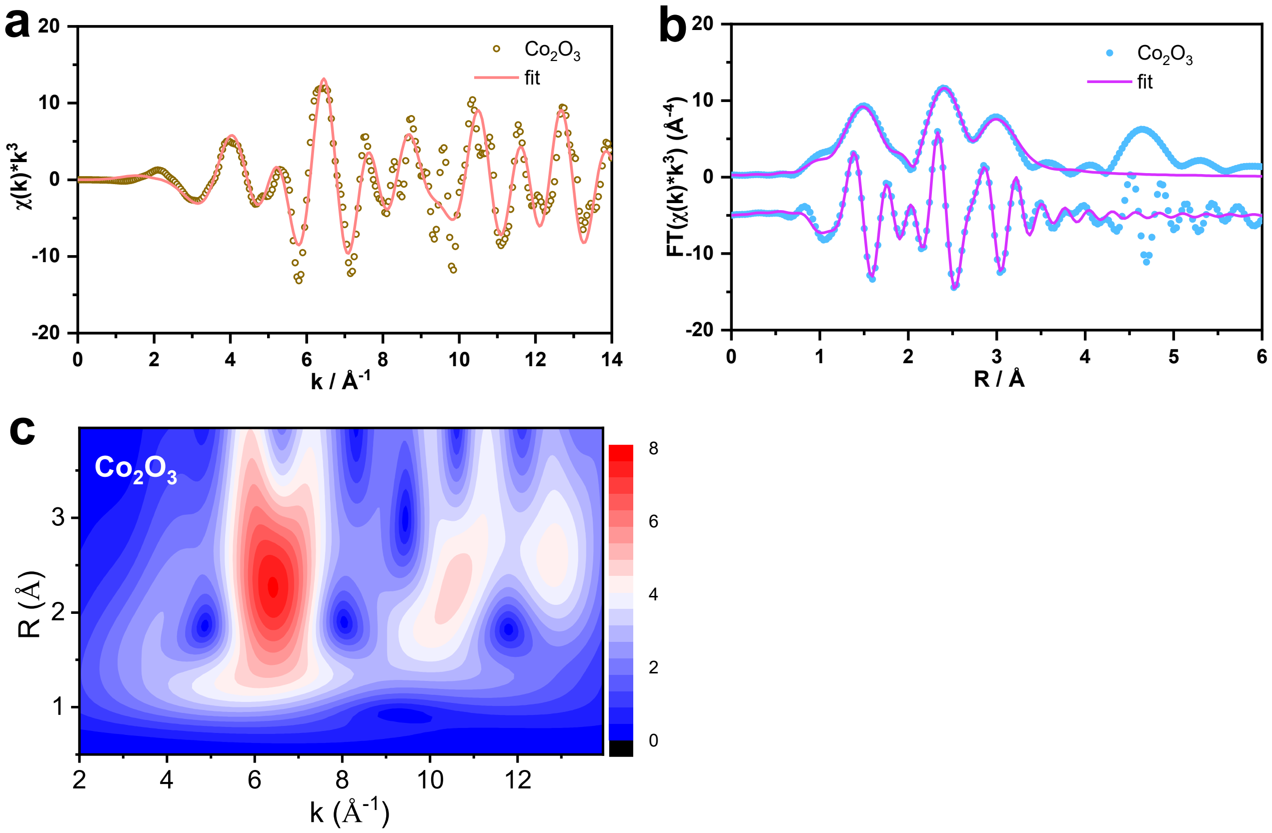

Supplementary Fig. 27. Fitting curves of the FT-EXAFS spectra for Co_2O_3: a, at K space. **b,** at R space. **c,** wavelet transform for the k^3-weighted EXAFS signals. Source data are provided as a Source Data file.

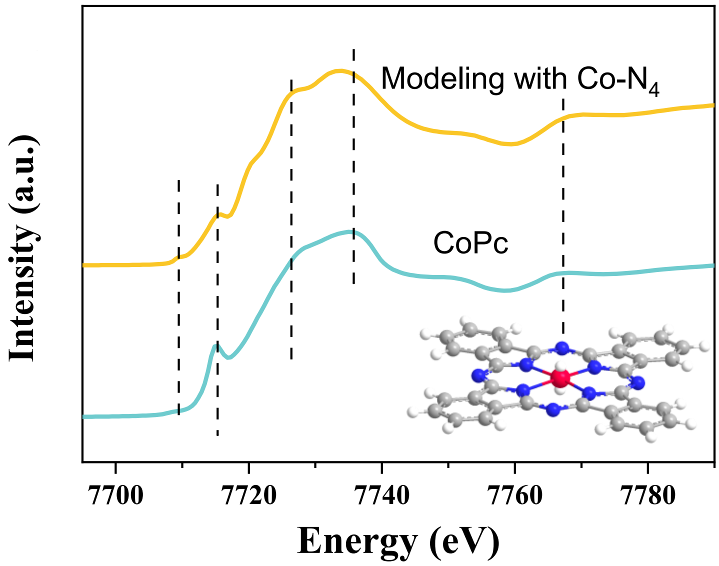

Supplementary Fig. 28. Comparison between the Co K-edge XANES experimental spectrum and the theoretical spectrum calculated with the depicted structure (inset) for CoPc. Source data are provided as a Source Data file.

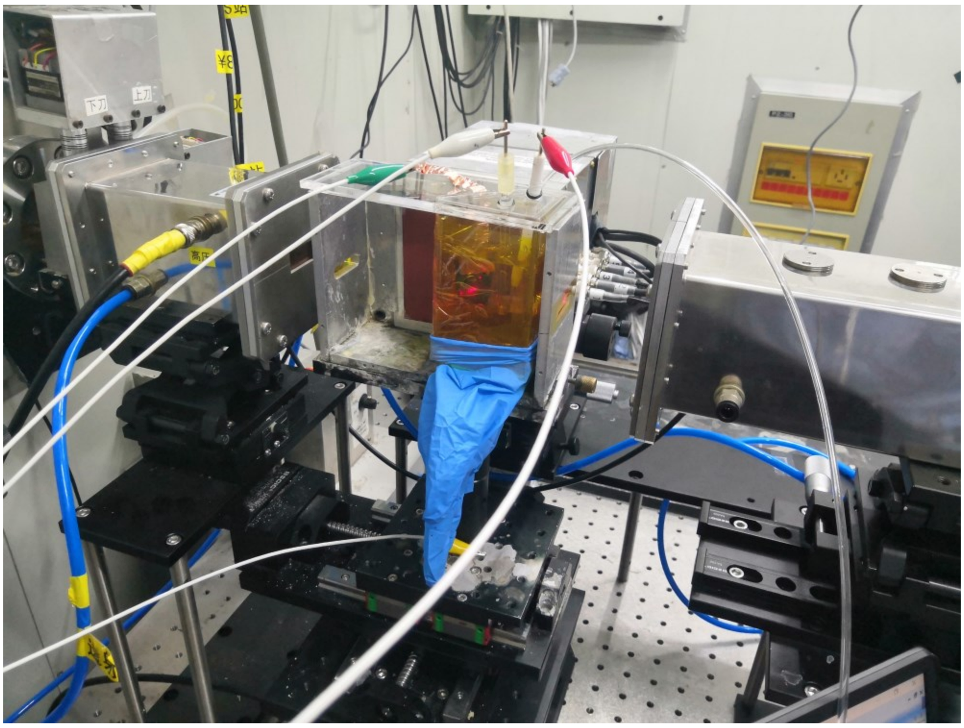

Supplementary Fig. 29. The operando XAS electrolyzer for electrochemical O_2 reduction.

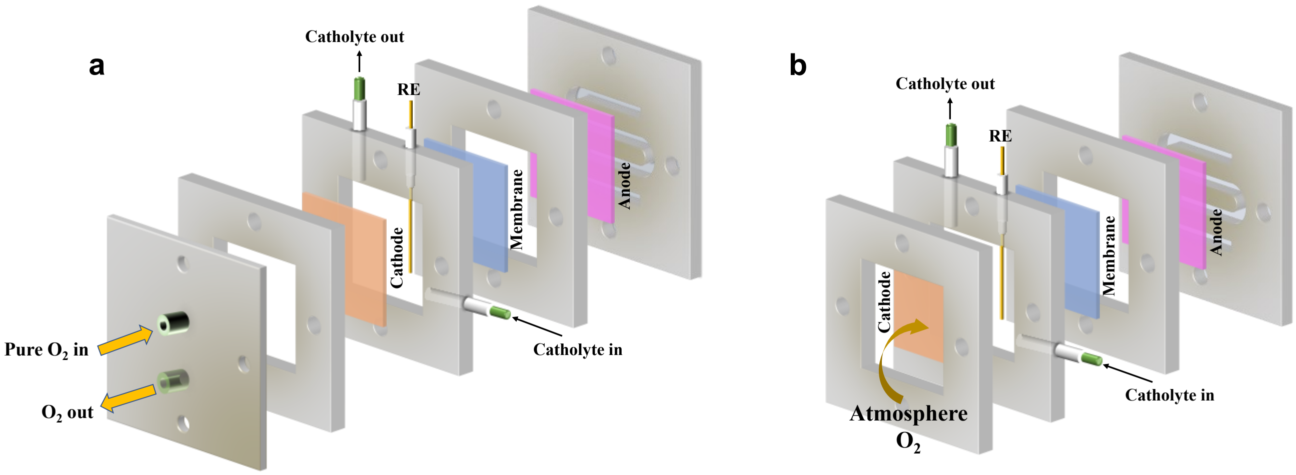

Supplementary Fig. 30. Schematic diagram of the air-breathing flow cell configuration: a, conventional flow cell purged with pure O_2 as the reactant. **b,** air-breathing flow cell with natural air diffusion electrode. Source data are provided as a Source Data file.

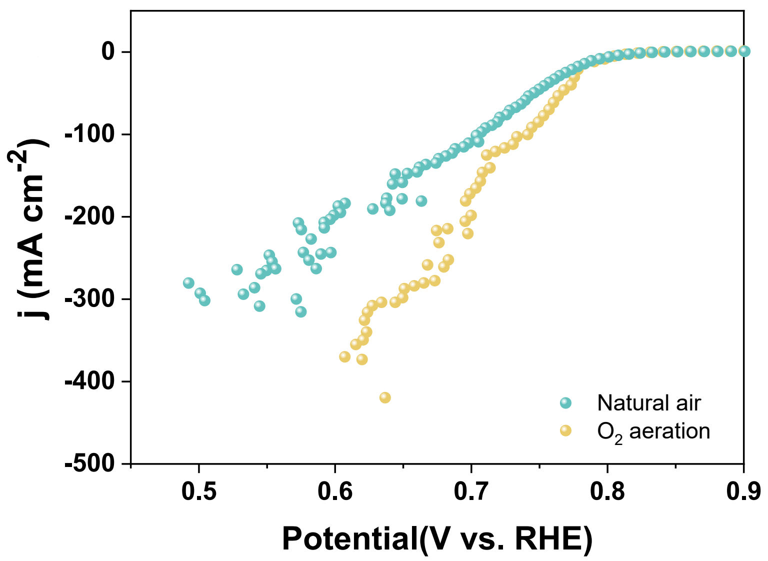

Supplementary Fig. 31. Comparison of the LSVs of Co HSACs in flow cell with O_2 aeration and air as the oxygen supply, respectively. The estimated cell voltage is ~6.5 V at 300 mA cm^{-2}. Source data are provided as a Source Data file.

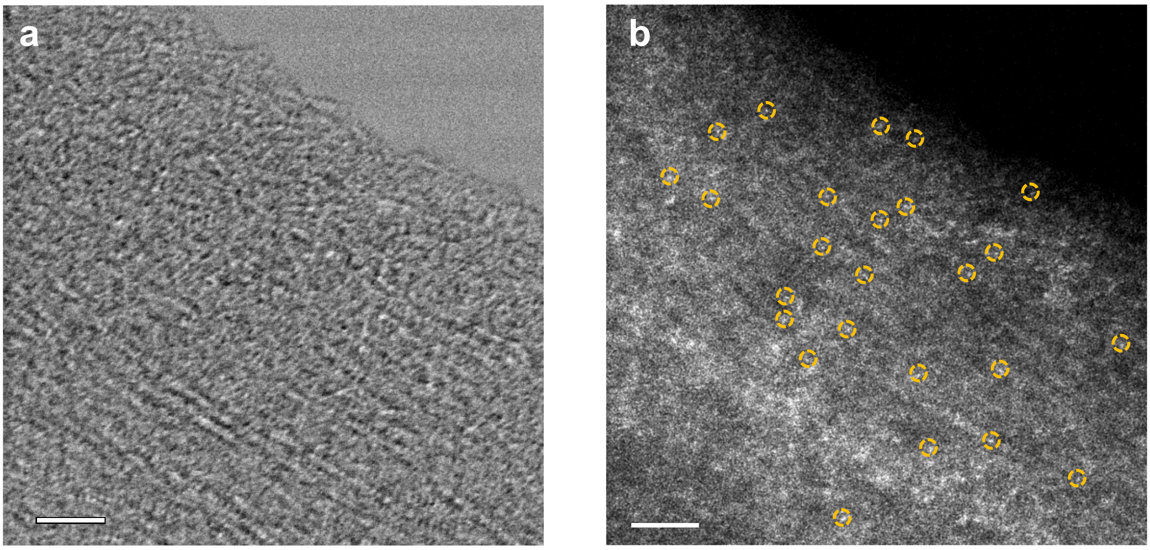

Supplementary Fig. 32. Aberration-corrected atomic-resolution TEM image (a) and HAADF-STEM image (b) of Co HSACs after stability test. The bright spots of Co atoms are marked with yellow circles. Source data are provided as a Source Data file.

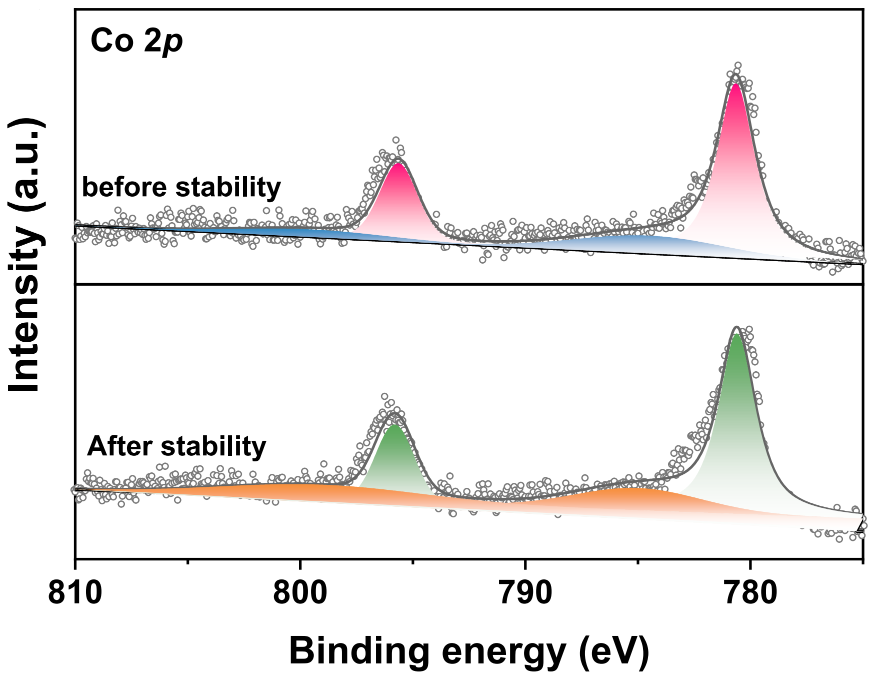

Supplementary Fig. 33. High-resolution XPS spectra of Co 2*p* for Co HSACs before and after the stability test. Source data are provided as a Source Data file.

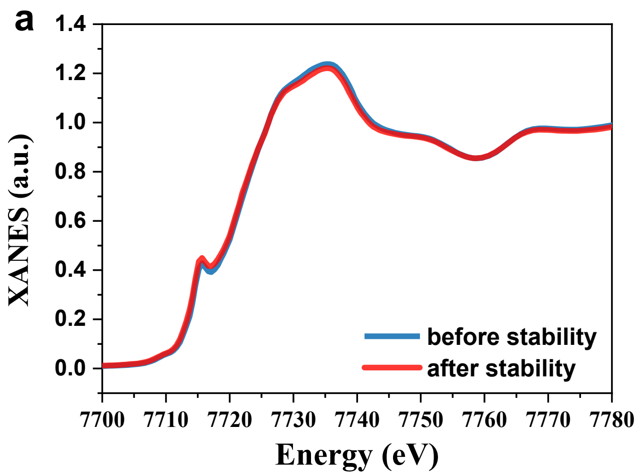

Supplementary Fig. 34. Comparison of the XANES and EXAFS spectra of the Co HSACs before and after stability test. Source data are provided as a Source Data file.

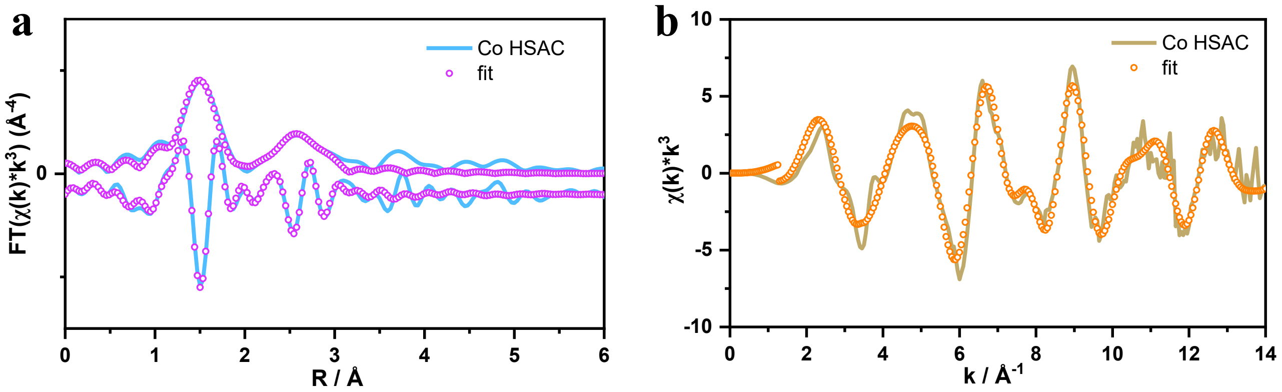

Supplementary Fig. 35. Fitting curves of the FT-EXAFS spectra for Co HSACs after stability: **a,** at R space. **b,** at K space. Source data are provided as a Source Data file.

Note:

As shown in Supplementary Fig. 31, the Co HSACs before and after stability test show nearly identical XANES spectra and EXAFS spectra. Furthermore, the coordination structure of Co-N was maintained after the stability test as displayed by the fitting curves of the FT-EXAFS shown in Supplementary Fig. 32 and Supplementary Table 9, suggesting the robustness of Co HSACs under high current density.

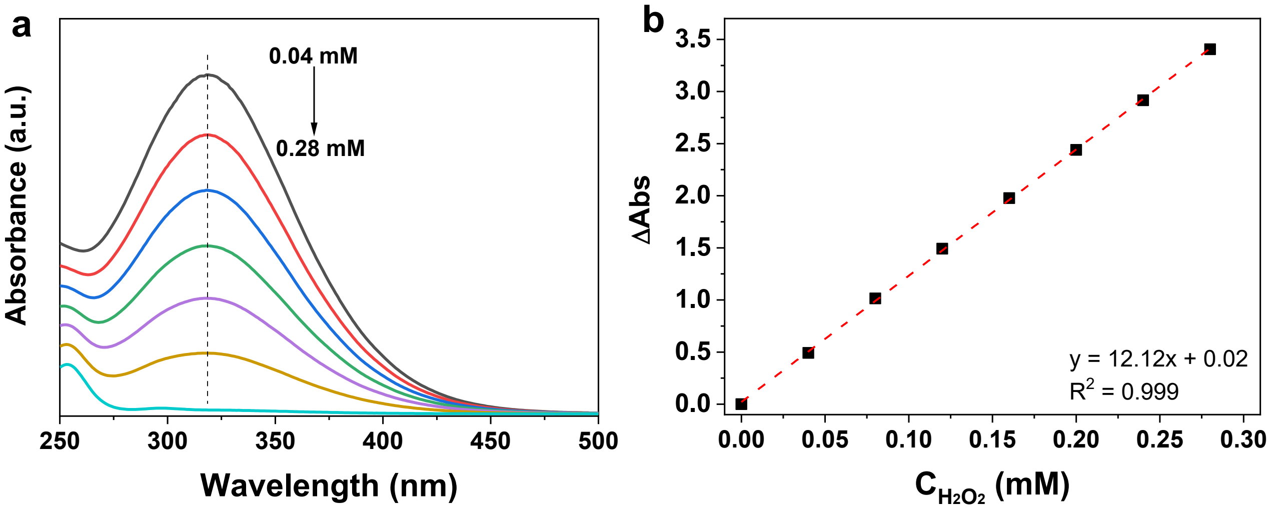

Supplementary Fig. 36. The H_2O_2 concentrations in electrolytes were measured by titration using standard ceric sulfate solution, $Ce(SO_4)_2$. (a) Absorption spectra changes of the cerium titration solution (1 mM) with the gradual addition of the H_2O_2 (0.04 - 0.28 mM). (b) Plot of the absorbance change at λ = 318 nm as a function of the concentration of H_2O_2. Source data are provided as a Source Data file.

Supplementary Table 1. Total energies and adsorption energies of ORR intermediates on MPc of various spin states.

		LS	ΔE_{LS}	IS	ΔE_{IS}	HS	ΔE_{HS}
TiPc	slab	-420.77					
	OH*	-432.79	-1.15				
	O*	-428.61	-0.34				
	OOH*	-437.03	2.12				
VPc	slab					-422.07	
	OH*					-433.37	-0.43
	O*					-429.67	-0.10
	OOH*					-437.65	2.80
CrPc	slab					-422.95	
	OH*					-433.39	0.68
	O*					-428.23	2.22
	OOH*					-437.68	3.64
MnPc	slab			-421.09		-421.58	
	OH*			-431.00		-431.90	0.56
	O*					-426.28	2.80
	OOH*			-435.30		-436.11	3.84
FePc	slab			-420.27		-420.02	
	OH*	-429.83	1.31	-430.48	0.66	-430.74	0.40
	O*	-424.58	3.19	-425.00	2.76	-424.92	2.85
	OOH*	-434.31	4.33	-434.71	3.93	-434.90	3.75
CoPc	slab	-419.64					
	OH*	-429.15	1.37	-428.43	2.08	-428.27	2.25
	O*	-423.56	3.58	-423.79	3.35	-423.67	3.47
	OOH*	-433.69	4.32	-432.79	5.22	-432.46	5.56
NiPc	slab	-417.18					
	OH*					-426.33	1.73
	O*					-420.41	4.27
	OOH*					-430.91	4.64
CuPc	slab	-416.26					
	OH*	-424.86	2.27				
	O*	-418.83	4.93				
	OOH*	-429.88	4.75				
ZnPc	slab	-415.67					
	OH*	-424.94	1.60				
	O*	-418.28	4.89				
	OOH*	-429.26	4.79				

Supplementary Table 2. Electrochemical parameters for the calculation of ECSA.

**Sample**	**Double-layer capacitance, C_{dl} (μF cm^{-2})**	**ECSA (cm^2)**
Pc	26.5	0.3242
MnPc	24.7	0.3056
FePc	26.2	0.3242
CoPc	22.1	0.2735
NiPc	25.2	0.3119
CuPc	27.7	0.3428
ZnPc	21.9	0.2710

Based on literature precedent, the double-layer capacitance of a planar glassy carbon surface, $C_{dl\text{-}planar}$, was taken as 20 μF cm^{-2}.

Supplementary Table 3. Total energies and adsorption energies of ORR intermediates on M HSACs of various spin states (M = Ti, V, Cr, Mn, Fe, Co, Ni, Cu, Zn).

		$\boldsymbol{E_{LS}}$/eV	$\boldsymbol{\Delta E_{LS}}$/eV	$\boldsymbol{E_{IS}}$/eV	$\boldsymbol{\Delta E_{IS}}$/eV	$\boldsymbol{E_{HS}}$/eV	$\boldsymbol{\Delta E_{HS}}$/eV
TiPc-py	slab	-492.96					
	OH*	-504.20	-0.37				
	O*	-500.09	0.37				
	OOH*	-508.38	2.95				
VPc-py	slab					-494.27	
	OH*					-505.15	0.00
	O*					-501.16	0.61
	OOH*					-509.45	3.19
CrPc-py	slab					-494.46	
	OH*					-505.36	-0.03
	O*					-499.92	2.04
	OOH*					-509.63	3.20
MnPc-py	slab	-491.86		-492.70		-493.52	
	OH*	-502.46	1.94	-503.00	1.39	-503.40	1.00
	O*	-497.37	3.65			-498.07	2.95
	OOH*	-506.71	5.19	-507.28	4.62	-507.65	4.25
FePc-py	slab	-491.45		-491.88		-491.29	
	OH*	-501.86	0.89	-501.99	0.76	-502.16	0.59
	O*	-496.40	2.98	-496.81	2.57	-496.51	2.87
	OOH*	-506.28	3.98	-506.25	4.00	-506.36	3.89
CoPc-py	slab	-491.33					
	OH*	-501.34	0.87	-500.67	1.53	-500.35	1.85
	O*	-495.58	3.25	-495.21	3.62		
	OOH*	-505.78	3.93	-505.18	4.52	-504.61	5.10
NiPc-py	slab	-488.65				-488.98	
	OH*					-497.81	2.05
	O*					-491.88	4.60
	OOH*					-502.60	4.75
CuPc-py	slab	-487.70					
	OH*					-496.34	2.24
	O*					-490.40	4.80
	OOH*					-501.31	4.76
ZnPc-py	slab	-487.47					
	OH*	-496.28	2.06				
	O*	-490.11	4.86				
	OOH*	-501.12	4.73				

Supplementary Table 4. Total energies and adsorption energies of ORR intermediates on M HSACs-F of various spin states (M = Ti, V, Cr, Mn, Fe, Co, Ni, Cu, Zn).

		E_{LS}/eV	ΔE_{LS}/eV	E_{IS}/eV	ΔE_{IS}/eV	E_{HS}/eV	ΔE_{HS}/eV
TiPc-pyF	slab	-493.11					
	OH*	-504.39	-0.40				
	O*	-500.28	0.33				
	OOH*	-508.62	2.87				
VPc-pyF	slab					-494.45	
	OH*					-505.34	-0.01
	O*					-501.37	0.58
	OOH*					-509.60	3.22
CrPc-pyF	slab					-494.69	
	OH*					-505.52	0.05
	O*					-500.12	2.07
	OOH*					-509.80	3.26
MnPc-pyF	slab	-492.05		-492.92		-493.29	
	OH*	-502.61	1.56	-503.15	1.02	-503.61	0.56
	O*	-497.41	3.38			-498.23	2.56
	OOH*	-506.86	4.81	-507.44	4.23	-507.84	3.83
FePc-pyF	slab	-491.62		-492.08		-492.22	
	OH*	-502.02	1.08	-502.19	0.91	-502.36	0.74
	O*	-496.57	3.15	-496.98	2.74	-496.68	3.03
	OOH*	-506.43	4.16	-506.44	4.15	-506.55	4.05
CoPc-pyF	slab	-491.55					
	OH*	-501.50	0.93	-500.88	1.55	-500.55	1.88
	O*	-495.74	3.36	-495.42	3.63		
	OOH*	-505.95	3.98	-505.38	4.55	-504.80	5.13
NiPc-pyF	slab	-488.83				-489.18	
	OH*					-498.00	2.06
	O*					-492.07	4.61
	OOH*					-502.80	4.75
CuPc-pyF	slab	-487.92					
	OH*	-496.51	2.29				
	O*	-490.58	4.84				
	OOH*	-501.53	4.77				
ZnPc-pyF	slab	-487.65					
	OH*	-496.51	2.01				
	O*	-490.35	4.80				
	OOH*	-501.28	4.75				

Supplementary Table 5. Total energies and adsorption energies of ORR intermediates on M HSACs-CH_3 of various spin states (M = Ti, V, Cr, Mn, Fe, Co, Ni, Cu, Zn).

		E_{LS}/eV	ΔE_{LS}/eV	E_{IS}/eV	ΔE_{IS}/eV	E_{HS}/eV	ΔE_{HS}/eV
TiPc-pyCH_3	slab	-509.63					
	OH*	-520.85	-0.35				
	O*	-516.74	0.39				
	OOH*	-524.93	3.08				
VPc-pyCH_3	slab					-510.95	
	OH*					-521.82	0.00
	O*					-517.82	0.63
	OOH*					-526.08	3.24
CrPc-pyCH_3	slab					-511.15	
	OH*					-522.02	0.00
	O*					-516.60	2.05
	OOH*					-526.30	3.23
MnPc-pyCH_3	slab	-508.51		-509.39		-509.78	
	OH*	-519.12	1.53	-519.65	1.00	-520.07	0.58
	O*	-514.02	3.26			-514.74	2.54
	OOH*	-523.39	4.76	-523.94	4.21	-524.32	3.83
FePc-pyCH_3	slab	-508.11		-508.56		-508.76	
	OH*	-518.51	1.13	-518.65	0.99	-518.81	0.83
	O*	-513.48	2.78	-513.47	2.77	-513.18	3.08
	OOH*	-522.94	4.19	-522.92	4.22	-523.02	4.12
CoPc-pyCH_3	slab	-508.02					
	OH*	-517.99	0.90	-517.34	1.55	-517.01	1.88
	O*	-512.24	3.28	-511.87	3.65		
	OOH*	-522.45	3.94	-521.84	4.55	-521.30	5.10
NiPc-pyCH_3	slab	-505.30				-505.67	
	OH*					-514.45	2.09
	O*					-508.48	4.69
	OOH*					-519.29	4.76
CuPc-pyCH_3	slab	-504.40					
	OH*	-512.99	2.28				
	O*	-507.04	4.86				
	OOH*	-517.97	4.80				
ZnPc-pyCH_3	slab	-504.16					
	OH*	-512.89	2.15				
	O*	-506.55	5.11				
	OOH*	-517.45	5.08				

Supplementary Table 6. XPS results for high-resolution N and Co for CNT and CNT-py.

**Catalyst**	**N content (at%)**	**Co content (at%)**
CNT	-	-
CNT-py	1.43	-
Co HSACs	6.15	0.64

Supplementary Table 7. Comparison of electrochemical H_2O_2 production via $2e^-$ ORR in alkaline solution.

Catalyst	Electrolyte	Onset potential (V, @0.1 mA cm^{-2})	Selectivity (%)	TOF (s^{-1})	Refs.
**Co HSACs**	**0.1 M KOH**	**0.85**	**~95**	**~0.89**	**This work**
**CoPc**	**0.1 M KOH**	**0.78**	**~80**	**~0.12**	**This work**
NiN_4/C-$AQNH_2$	0.1 M KOH	0.83	~80	~0.12	Advanced Materials, 2104891 (2021).
Co_1-NG(O)	0.1 M KOH	~ 0.83	~ 80	~2.70	Nat. Mater. 19, 436-442 (2020).
Fe-CNT	0.1 M KOH	0.822	~ 95	~0.57	Nat. Commun. 10, 3997 (2019)
Co-CNT	0.1 M KOH	~ 0.80	~ 75	~0.34	Nat. Commun. 10, 3997 (2019)
Co-POC-O	0.1 M KOH	0.84	~ 84	~0.21	Adv. Mater. 31, 1808173 (2019)
Oxidized CNTs	0.1 M KOH	0.75	~ 89	~0.0178	Nat. Catal. 1, 156-162 (2018)
F-mrGO	0.1 M KOH	0.78	95	~0.088	Nat. Catal. 1, 282-290 (2018)
O-GOMC	0.1 M KOH	~0.82	~95	~0.10	Chem. 7(11): 3114-3130 (2021).

Supplementary Table 8. Co K-edge EXAFS spectra fitting parameters.

**Sample**	**Path**	**CN**	**R (Å)**	**σ^2 ($\times10^{-3}$ Å^2)**	**ΔE_0 (eV)**	**R (%)**
Co foil[a]	Co-Co	12	2.50 ± 0.01	6.3	7.90 ± 0.27	0.05
CoPc[b]	Co-N_1	4.1	1.91 ± 0.01	3.0	5.12 ± 1.63	0.6
	Co-C	8.9	2.94 ± 0.02	3.0		
	Co-N_2	4.5	3.26 ± 0.02	3.0		
Co HSACs[c]	Co-N_1	5.0	1.92 ± 0.01	2.8	5.35 ± 1.12	0.6
	Co-C_1	27.3	2.98 ± 0.01	13.4		
	Co-N_2	15.5	3.26 ± 0.01	8.0		
	Co-C_2	5.9	3.99 ± 0.03	3.0		
	Co-C_3	24.5	4.28 ± 0.02	10.0		

N, coordination number; R, distance between absorber and backscatter atoms; σ^2, Debye-Waller factor to account for both thermal and structural disorders; ΔE_0, inner potential correction; R factor (%) indicates the goodness of the fit. S_0^2 was fixed to 0.76 as determined from Co foil fitting.

[a] Fitting range: $3.3 \leq k$ (/Å) ≤ 11.9 and $1.0 \leq R$ (/Å) ≤ 2.7.

[b] Fitting range: $2.7 \leq k$ (/Å) ≤ 11.5 and $1.1 \leq R$ (/Å) ≤ 3.3.

[c] Fitting range: $2.7 \leq k$ (/Å) ≤ 11.5 and $1.0 \leq R$ (/Å) ≤ 4.0.

Supplementary Table 9. Co K-edge EXAFS spectra fitting parameters of Co HSACs before and after stability.

**Sample**	**Path**	**CN**	**R (Å)**	**σ^2 ($\times10^{-3}$ Å^2)**	**ΔE_0 (eV)**	**R (%)**
Co HSACs before stability	Co-N	5.0	1.92 ± 0.01	3.0 ± 1.0	6.81 ± 1.63	1.9
Co HSACs after stability	Co-N	4.9	1.90 ± 0.01	3.0 ± 1.0	6.03 ± 1.96	1.8

N, coordination number; R, distance between absorber and backscatter atoms; σ^2, Debye-Waller factor to account for both thermal and structural disorders; ΔE_0, inner potential correction; R factor (%) indicates the goodness of the fit. S_0^2 was fixed to 0.82 as determined from Co foil fitting.

[a] Fitting range: 2.7 ≤ k (/Å) ≤ 11.5 and 1.0 ≤ R (/Å) ≤ 4.0.

[b] Fitting range: 2.7 ≤ k (/Å) ≤ 12.3 and 1.0 ≤ R (/Å) ≤ 3.3.

Supplementary Note 1

Discussion on the equilibrium potential for 2e⁻ pathway ORR in alkaline media

The CoPc-py-CNT HSAC exhibit the onset potential for H_2O_2 production at ~0.85 V vs. RHE, which is higher than the onset potential of thermodynamic limit of 0.76 V vs. RHE in alkaline solution. In acidic solution, the pK_a values for the first and second ionization of H_2O_2 at 298 k are 11.69 (pK_{a1}) and ~20 (pK_{a2}), respectively. Therefore, the predominant hydrogen peroxide species at pH < 12 and pH > 12 are H_2O_2 and HO_2^-, respectively. In alkaline media, HO_2^- is the deprotonated form of H_2O_2. During the LSV test, the more positive onset potential than the thermodynamic limit of 0.76 V is attributed to the Nernst related potential shift due to the relatively low concentration of H_2O_2 in the electrolyte[1].

$$O_2 + 2H_2O + 4e^- \rightarrow 4OH^- \qquad E_o = 1.23 \text{ V vs. RHE} \qquad (1)$$

$$O_2 + H_2O + 2e^- \rightarrow HO_2^- + OH^- \qquad E_o = 0.76 \text{ V vs. RHE} \qquad (2)$$

References

1. H. W. Kim, M. B. Ross, N. Kornienko, L. Zhang, J. Guo, P. Yang and B. D. McCloskey, *Nat. Catal.*, 2018, **1**, 282-290.
